# CRISPR-Analytics (CRISPR-A): A platform for precise analytics and simulations for gene editing

**DOI:** 10.1371/journal.pcbi.1011137

**Published:** 2023-05-30

**Authors:** Marta Sanvicente-García, Albert García-Valiente, Socayna Jouide, Jessica Jaraba-Wallace, Eric Bautista, Marc Escobosa, Avencia Sánchez-Mejías, Marc Güell

**Affiliations:** 1 Department of Medicine and Life Sciences, Universitat Pompeu Fabra, Barcelona, Spain; 2 Faculty of Mathematics and Computer Science, University of Barcelona, Barcelona, Spain; 3 Integra Therapeutics S.L., Barcelona, Spain; 4 Department of Health Technology, Technical University of Denmark, Lyngby, Denmark; ., CANADA

## Abstract

Gene editing characterization with currently available tools does not always give precise relative proportions among the different types of gene edits present in an edited bulk of cells. We have developed CRISPR-Analytics, CRISPR-A, which is a comprehensive and versatile genome editing web application tool and a nextflow pipeline to give support to gene editing experimental design and analysis. CRISPR-A provides a robust gene editing analysis pipeline composed of data analysis tools and simulation. It achieves higher accuracy than current tools and expands the functionality. The analysis includes mock-based noise correction, spike-in calibrated amplification bias reduction, and advanced interactive graphics. This expanded robustness makes this tool ideal for analyzing highly sensitive cases such as clinical samples or experiments with low editing efficiencies. It also provides an assessment of experimental design through the simulation of gene editing results. Therefore, CRISPR-A is ideal to support multiple kinds of experiments such as double-stranded DNA break-based engineering, base editing (BE), primer editing (PE), and homology-directed repair (HDR), without the need of specifying the used experimental approach.

## Author summary

Precision and accuracy are paramount for genome editing usage in sensitive applications such as clinical. This field requires reliable and traceable results to ensure the safety and viability of the resulting genomic modifications. To go in this direction, we have developed a computational platform that can simulate and analyze genome editing outcomes comprehensively and in a versatile manner. Our computational pipeline and web application, CRISPR-Analytics (CRISPR-A), expands the functionality of currently available tools. The added robustness of our tool makes it perfect for analyzing sensitive data, such as clinical samples or experiments with low editing efficiencies.

This is a *PLOS Computational Biology* Methods paper.

## Introduction

CRISPR-based gene editing has become a fundamental toolbox to cover a large variety of research and applied needs. It facilitates the editing of endogenous genomic loci and systematic interrogation of genetic elements and causal genetic variations [1–3]. Nowadays, it is even on the verge of becoming a therapeutic reality *in vivo* [4]. Despite tremendous advances, DNA editing and writing still involve imperfect protocols which need to be optimized and evaluated. This makes it essential to have tools that enable accurate characterization of gene editing outcomes.

Gene editing outcomes often involve complex data sets with a diverse set of genotypes. This is especially accentuated for double-stranded DNA-based gene editing such as those based on non-homologous end-joining (NHEJ) or homology directed repair (HDR). These experiments often generate complex gene editing signatures involving insertions, substitutions, and deletions. Accurate quantification of this distribution of genotypes may have important implications including knockout integrity or splicing modulation. On one hand, experimental conditions, gene editing reagents, and cell types influence gene editing outcomes. Analytical tools, and simulations are needed to help in the experimental design. DNA repair outcomes have incomplete predictability [5], and deep exploration of DNA repair pathways shows that double strand break (DSB) repair gene mutations with similar sequences can come from different repair mechanisms [6]. Unraveling this complexity can help in the development of editing tools with higher efficiency and specificity, as well as in the implementation of better prediction models. In addition, when certain genotypes have to be enriched, prediction is even more relevant in design. One example is HDR improvement by targeting indel byproducts, where the prediction of the outcomes of the first target editing, allows the design of a second gRNA, that targets the more probably abundant indel outcome of the first gRNA, to give a second chance to HDR [7]. On the other hand, a precise gene editing assessment is essential for validation purposes.

Initial methods for editing assessment were based on T7 endonuclease 1 (T7E1) mismatch detection assay and Surveyor Mismatch nucleases enzyme assays, which are based on the detection of DNA heteroduplexes after PCR amplification, denaturalization, and reannealing. When editing takes place, the different reannealed products generate heteroduplexes, which are cleaved by T7E1 [8]. Tracking of Indels by Decomposition (TIDE) is another method with the same purpose. TIDE is based on PCR amplification, capillary sequencing and deconvolution algorithm [9]. Indel Detection by Amplicon Analysis (IDAA) requires a tri-primer amplification labeling and DNA capillary electrophoresis for indel detection [10]. Another genotyping method that has been successfully used to evaluate genome editing is high-resolution melting analysis (HRMA) [11]. This is a simple and efficient real-time polymerase chain reaction-based technique. These methods are generally cheap and quick on execution but fail to provide a comprehensive report of the gene editing outcome. Finally, Next Generation Sequencing (NGS) methods have also been broadly used to characterize gene editing efficiency [12]. NGS techniques are the optimal platforms for accurate quantification of indel size, frequency, and sequence identity determination [13]. The analysis of NGS results requires the development of bioinformatic algorithms. Some tools developed for the genome editing assessment include CRISPR-GA [14], CRISPResso [15], CrispRVariants [16], CasAnalyzer [17], cris.py [18], CRISPResso2 [19], ampliCan [20] and CRISPRpic [21]. Other tools, like CRISPRnano, are focused on the analysis of Oxford Nanopore Technologies (ONT) data, instead of NGS data sequenced by synthesis [21, 22]. Moreover, a new analysis tool, CRISPECTOR, has recently been developed to cover specificity assessment related to off-targets and translocations [23].

However, these tools have some limitations (S1 Table). For instance, quantification of different outcomes is poorly or imprecisely reported, these tools do not include simulations to help in design or benchmarking; the frequencies are based on absolute counts without taking into account amplification and sequencing biases. Most of these tools lack important functionalities like reference identification, clustering sequences by Unique Molecular Identifiers (UMIs), or noise subtraction. Preferential clustering of smaller fragments by NGS technologies has been previously shown [24], as well as sequencing error rates [25]. Even not all of them have the same missing functionalities, as can be seen in the S1 Table, CRISPR-A is the only tool that can identifies the amplicon reference from a reference genome, correct errors through UMI clustering and sequence consensus, correct quantification errors due to differences in amplicon size, and includes interactive plots and a genome browser representation of the alignment.

In this context, CRISPR-A emerges as an evolution of CRISPR-GA, the first NGS-based method for gene editing assessment. CRISPR-A simulated gene editing data takes into account the different repair mechanisms involved in DBS repair, and it is capable of analyzing edited bulk cell populations as well as individual clones. To address previous limitations, we have explored alignment and variant calling methods to overcome the challenges of a precise characterization. The operation of different alignment methods, the implementation of different filters, and the correction of sequencing bias are studied to reach an accurate quantification of editing events. Development and benchmarking of CRISPR-A have been done using simulated data as well as a big variety of targeted NGS data, and even human validated data. From CRISPR-A functionalities we highlight: (i) the development of a simulator together with the analysis pipeline, (ii) calibrated alignment method and new variant caller aware of the targeting site, (iii) batch samples analysis, (iv) nextflow pipeline implementation (https://bitbucket.org/synbiolab/crispr-a_nextflow/) that is being also implemented in nf-core (https://nf-co.re/crisprseq), and (v) a web application with a user friendly and interactive interface (https://synbio.upf.edu/crispr-a/). In addition, it includes reports and visualizations of indels (including micro-homology patterns), substitutions and objective modification quantification, and in frame and out of frame indels report. Finally, we want to highlight the enhanced precision using Unique Molecular Identifiers (UMIs) and spike-in controls, as well as noise reduction with an empirical model based on negative control samples (mocks). Precision will be key for applications that require accurate and traceable results such as clinical CRISPR applications and environmental or industrial uses.

## Results

### Gene editing simulations provide design assessment

**Fig 1:**
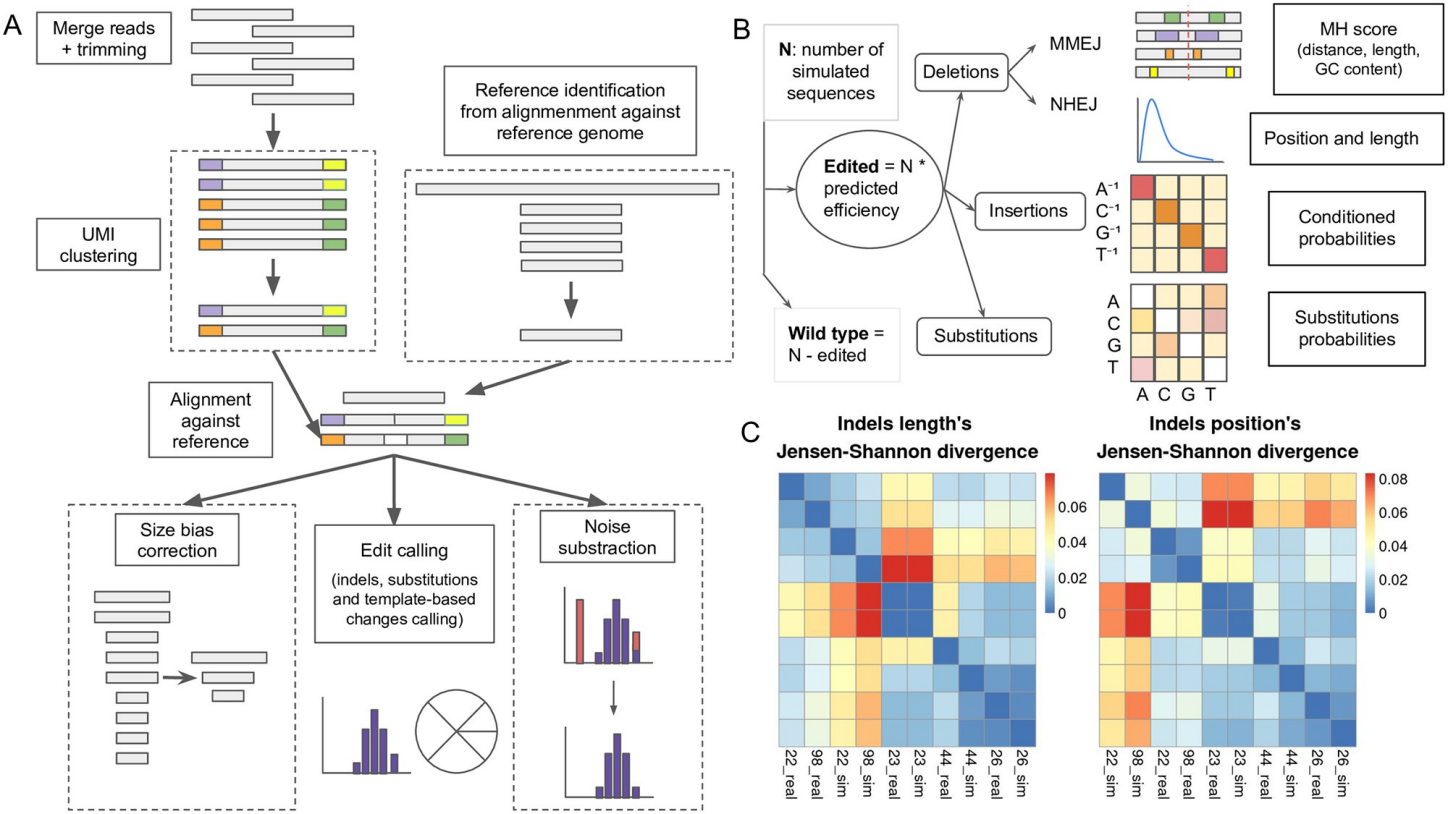
CRISPR-A capabilities for analysis and simulation of CRISPR-based experiments. A) Diagram of CRISPR-A analysis pipeline. The analysis algorithm is composed of three mandatory steps: reads pre-processing for quality assessment, reads alignment against reference amplicon, and edit calling. The processes inside dashed line squares are optional, being: UMI clustering, reference discovery, size bias correction, and noise subtraction based on an empirical model from negative control samples. B) Diagram of CRISPR-A simulation pipeline. Simulation is based on the fitting of multiple parameters that describe the distribution of edits and their characteristics and proportions. Once the number of edited sequences is determined by the protospacer predicted efficiency, other probability distributions are applied to decide the number of each kind of edit (NHEJ deletions, MMEJ deletions, insertions, and substitutions). C) Heatmap to visualize hierarchical clustering of real samples and their simulations from the validation data set. The clustering distance used is the JS divergence between the two subsets. Data values are transformed to the color scale depicted on the right. Dark blue equals 0 distance and identical samples, while red is for the greatest distance value. The plot on the left shows the distances between the position of the variant and the cut site, while the plot on the right is for the size of the variants. For these plots, we have randomly selected 5 pairs of real samples from the T cells validation data set (k = 100): SRR7737126, SRR7737722, SRR7737698, SRR7736744, and SRR7736723. We have labeled the samples with the two last numbers of the sample name adding real for the real sample and sim for its simulation. For instance, SRR7737722 and SRR7737698, which cluster together, are the real sample and its simulated sample for two replicates.

We developed CRISPR-A, a gene editing analyzer that can provide simulations to assess experimental design and outcomes prediction. These simulations are generated by SimGE, an R package that, for ease of use, is implemented within the CRISPR-A platform (Fig 1A). This algorithm is useful to generate simulated data of edited reads for CRISPR analysis tools evaluation as well as for design purposes. The SimGE algorithm is based on the characterization of repair outcomes in primary T cells [25, 26], which is a promising cell type for therapeutic ex vivo genome editing. It simulates repair outcomes of CRISPR-Cas9 knockout experiments and it is able to simulate the most common variants: insertions, deletions, and substitutions, based on observed experimental data edit distributions (Fig 1B). Same parameters and probability distributions were fitted for three other cell lines: Hek293, K562, and HCT116 [27], to make SimGE more generalizable and increase its applicability.

From the characterized data, we obtained the probability distribution of each class along with the individual DNA repair outcomes, deletions, and insertions taking into account sequence context, mutation efficiencies, and single-nucleotide variants. It was found that gamma distribution fitted best for both the insertions and substitutions whereas the beta distribution described better the size and location of the deletions. For insertions, we have observed that the majority of them, 91.79%, are 1bp (S1A and S1B Fig), thus, SimGE predicts just insertion of 1bp size. Deletion modification sizes, as well as starting position distributions of all kinds of edits, are defined from experimental data. This ensures that the simulations are distributed in a comparable way to what we would expect after a CRISPR-Cas9 knockout experiment. After comparing the deletion and insertion size distribution from two different data sets, we observed that indel variants decrease quickly as the size increases (S1A and S1B Fig), being consistent with previous observations [28].

Among the evaluated metrics, Jensen distance showed the best clustering performance between real samples and replicates and, as expected, a greater separation with the unrelated samples (S2 Fig). For this reason, this is the metric used to compare indel sizes and start positions between experimental samples and simulations. After testing the different combinations of Euclidean distances (mean, median and quantiles; see Methods), we see that all three show a very similar clustering pattern.

To illustrate the results of the evaluation of the model, we have selected a subset of 5 experimental samples with their equivalent simulated samples. The clustering is depicted in Fig 1C. Using Jensen distance, we see that each pair of experimental and simulated samples show a lower distance between them in all 5 different cases, compared to samples with different targets. In addition, on top of comparing the distance between the experimental sample and their simulated one, we have included two experimental samples, SRR7737722 and SRR7737698, which are experimental replicates. These two and their simulated samples show a low distance between them and a higher distance with other samples.

### CRISPR Analytics: A versatile and intuitive tool for genome editing simulation and analysis

CRISPR-A analyzes a great variety of experiments with minimal input. Single cleavage experiments, base editing (BE), prime editing (PE), predicted off-target sites or homology directed repair (HDR) can be analyzed without the need of specifying the experimental approach. Also, if the amplified genome is given, the specification of the reference amplicon sequence can be avoided, as the tool will identify which sequence must be used as an amplicon reference. Therefore, the CRISPR-A pipeline just requires NGS targeted data to compute gene editing results. Even single-molecule sequencing (eg. PacBio, Nanopore…) can be analyzed by CRISPR-A, targeted sequencing by synthesis data is required for precise quantification. Amplicon sequence, protospacer sequence, and cut site position relative to the protospacer, and template are other optional inputs.

The first step of the pipeline is the quality control of Next Generation Sequencing (NGS) raw data, which consists of merging forward and reverse reads and trimming short and low quality sequences, as well as adapter sequences. When Unique Molecular Identifiers (UMIs) are used to increase precision, reads are clustered by these sequences, and the consensus of the cluster is used in the following steps. The next step is the alignment of the reads against the amplicon sequence, which is used in the same orientation as the protospacer to achieve comparable results. After that, custom scripts have been developed for CRISPR edits calling, quantification size bias correction by spike-in controls, and mock-based noise subtraction. Finally, several interactive tables and plots are generated to visualize the results (Fig 1A).

To standardize the analysis, CRISPR-A pipeline is implemented with Nextflow, a workflow management system that uses Docker technology for containerized computation [29]. First, NGS data can be uploaded as fastq files or it can be simulated with SimGE. Second, a reference amplicon can be directly uploaded or, in the case of an available reference genome, the reference amplicon can be identified by aligning the reads against the reference genome. Third, the protospacer sequence is also required for further standardizations in the analysis process and visualization (S3A Fig). SimGE, the genome editing simulator that we have assessed using five-fold cross-validation (S3B Fig), is also part of the pipeline. In the case of simulating edits, cell lines as well as sequence context is taken into account to determine the proportion of each outcome (S3C Fig). On top of the pipeline, CRISPR-A has a user-friendly and interactive web application to visualize all the results provided by the analysis. A genome browser is included to visually evaluate the performance of the alignment. Samples can be easily compared by summary tables and heatmaps. Interactive pie plots show the count of reads in all pre-processing steps, indels characterization, and editing. Other relevant information, such as micro-homology patterns leading to deletions, substitutions, and more abundant alleles, are also shown graphically. CRISPR-A does not make assumptions during the analysis, hence, the cut site is not used for filtering as other tools use (e.g. CRISPResso2, CRISPRpic, CRISPECTOR…). Nevertheless, the user has the option of refining the percentage of editing indicating the desired filtering windows iteratively once the results are shown (S1 Video).

### CRISPR-A effectively calls indels in simulated and edited samples

Gene editing simulations obtained with SimGE were used to develop the edits calling algorithm as well as for benchmarking CRISPR-A with other tools that have similar applications. The name of each simulated edit indicates its type (insertion, deletion or substitution) and characteristics for an easier further validation of the edits classification.

**Fig 2:**
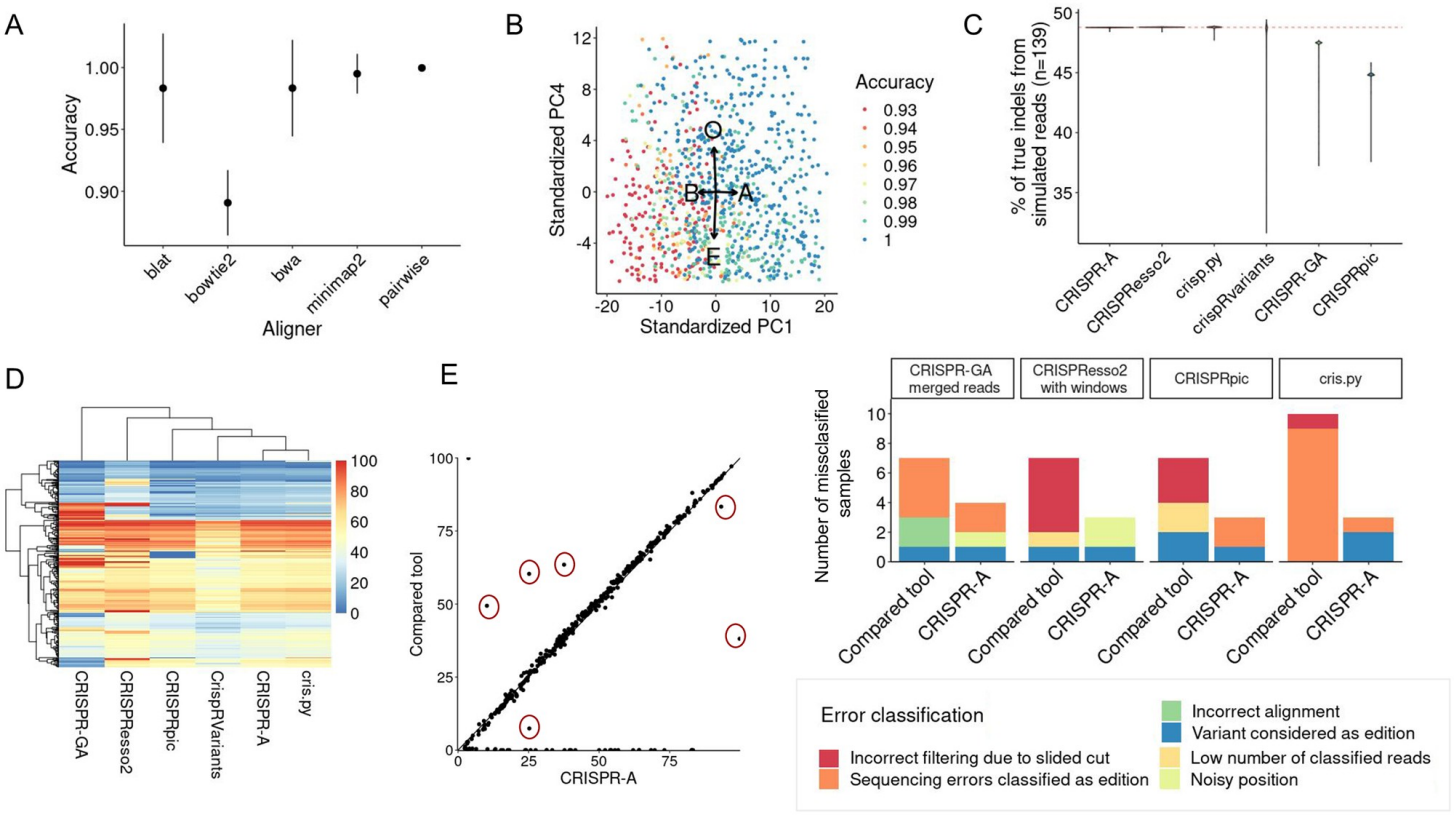
Indels characterization algorithm development and benchmark with simulated data and real data. A) Accuracy of indels detection after aligning with different alignment tools. We have used 139 samples simulated from different reference sequences and cut site location with SimGE. The accuracy, which is the number of reads correctly classified against all classified reads, has been calculated after characterizing the indels and wt sequences of each sample after aligning the samples with 6 different methods. The mean and standard deviation of the accuracy of the different characterized samples by alignment method are represented in the plot. B) Optimization of alignment penalty matrix. Best parameters have been determined through Monte Carlo to obtain alignments optimal for CRISPR-based indels characterization. In the PCA we can see how the four parameters of the alignment penalty matrix should be combined to achieve higher accuracy. C) Benchmarking of indels characterization between 6 different tools. CRISPR-A is compared with 5 other tools using a simulated data set of 139 samples. All samples contain the same percentage of indels (red dashed line) and the violin plot shows us the dispersion of the reported editing percentages by each tool. D) Reported editing of edited t-cells. 1656 unique edited genomic locations within 559 genes are characterized with 6 different tools. The percentage of editing reported by each tool for each sample is shown by the heatmap. E) Error characterization from the most discrepant values in t-cell edited samples. CRISPR-A results are compared with the results of other tools with more distant results (example at left side; explored samples are encircled in red). Errors are classified regarding their source.

Different alignment methods were compared to establish the best method to characterize CRISPR-based indels (Fig 2A). Most of the mischaracterized alignments, for all compared alignment tools, were due to multiple shorter indels, instead of a single deletion or insertion, which is the expected result of a DSB repair (S4 Fig). Although all alignment methods make this kind of error, BWA-MEM [30] is the one showing the highest abundance.

Nevertheless, we have also reported other kinds of errors. For instance, Bowtie2’s [31] main error type is the lack of alignment, while BLAT [32] is more prone to mislead the type of change. Pairwise alignment and minimap2 [33] show similar performance. Thus, since minimap2 is as well based on pairwise alignment and is ten-fold faster, we have focused on improving CRISPR variant alignment results with minimap2. Therefore, we have performed a Monte Carlo analysis with a random sampling of alignment parameters to increase the accuracy. Alignment and edit calling of the simulated samples were done with the parameters obtained from 950 iterations of a random selection of alignment matrix penalty values (matching score (A), mismatching score (B), gap open penalty (O), and gap extension penalty (E)). After performing a Principal Component Analysis (PCA), Fig 2B shows a high relevance of high values in A to achieve high accuracy values, while low values of B help to obtain higher accuracies. Furthermore, high values of O and low values of E can be observed in samples characterized by high accuracy.

To evaluate the improvements brought by the optimization of the aligner, our method is benchmarked with other currently available tools (Fig 2C). CRISPR-A is as good as CRISPResso2 [19] in characterizing simulated data as both tools characterize almost all samples with a good approximation of indels percentage. CRISPRpic [34] has the highest mean distance to the true value (-4.03%), followed by CRISPR-GA (-2.58%) [14]. CrispRvariants [16], even with a mean closer to the expected value, has the highest standard deviation (3.09%).

To further explore the different tools and their performance, we compared them using targeted NGS data analysis from the primary T cells data set [26]. When we compare the reported percentage of editing of all samples by the same 6 different tools, we see that CRISPR-A and cris.py show similar results. CrispRVariants is the one with lower reported percentages, and CRISPResso2 and CRISPR-GA have the higher amount of extreme values (Fig 2D). To make CRISPResso2 and CRISPR-GA results more comparable with the other tools, we have given the pair-end reads assembled with PEAR to CRISPR-GA, and the protospacer to filter by quantification window to CRISPResso2. Then, we see a reduction of extreme values in CRISPResso2, and a reduction of dispersed values in CRISPR-GA (S5 Fig). After that, we also compared the editing percentage reported by all these tools against the indels percentage given by CRISPR-A. CRISPRpic shows a high number of samples with null editing percentage, while other samples are giving editing values for these same samples. CrispRVariants shows, in general, lower reported percentages compared with the other tools, and a higher dispersion for indels with higher editing (S5 Fig). In addition, we explored more in detail the samples with the highest difference of reported percentage of editing compared with our tool (S2 Table). After comparing in detail these samples we can conclude that CRISPRpic and CRISPResso2 tend to report single nucleotide polymorphisms (SNPs) as edits and remove true indels produced by CRISPR systems when the cut site is slided, while CRISPR-A and cris.py tend to report error prone sequence regions as edits (Fig 2E). We have improved CRISPR-A to avoid these errors. There are 6 samples in which CRISPR-A has been considered to be giving an incorrect editing percentage report. There is one sample, SRR7736645, whose amplicon sequence does not coincide with the one of the reference genome. It has an heterozygous variant, an inversion in one allele, and an insertion in the same position in the other allele (S6 Fig). Since the mutation is not close to the cleavage site, in this case, the tools that apply a filtering window are the ones giving a better approximation of the editing. Instead of filtering edit events by windows, we have implemented a process in CRISPR-A to ignore indels in certain regions (variants or error prone sequences). After that, CRISPR-A gives an editing percentage comparable to the results of those tools that use a quantification window in the cut site. Samples SRR7736865, SRR7737875, and SRR7737569 have been analyzed with a sequence shorter than the amplicon sequence and an incorrect protospacer. For this reason, all samples are reporting a lower percentage of editing. Once the reference sequence used is the one corresponding to the whole reference amplicon, obtained with CRISPR-A amplicon sequence discovery function, CRISPR-A shows a perfect editing profile (S7 Fig). Finally, samples SRR7736598 and SRR7736646 have a polyadenine sequence of length 14 that is poorly sequenced (S8A Fig). For this reason, the tools with filtering windows have a better estimation of the editing. When there is no negative control for noise subtraction, CRISPR-A automatically searches repetitive sequences and ignores indels found in those regions. After including this new functionality, CRISPR-A is able to correctly classify indels and wild type sequences in error prone sequences (S8B Fig).

### Improved discovery and characterization of template-based alleles or objective modifications

**Fig 3:**
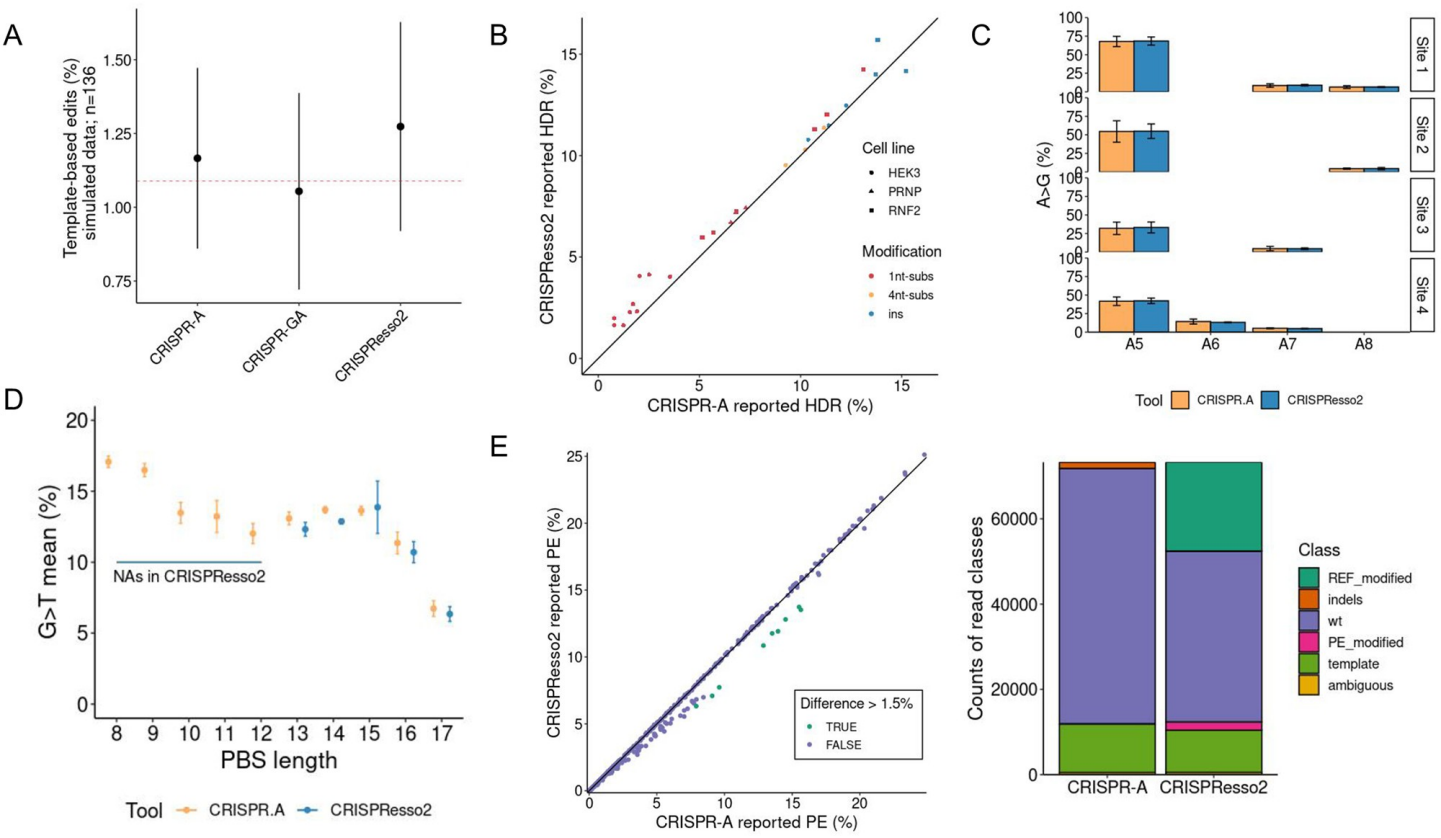
Template-based edits and substitutions characterization benchmark with simulated data and real data. A) Benchmarking of template-based editing characterization between 3 different tools. CRISPR-A is compared with 2 other tools using a simulated data set of 136 samples. All samples contain the same percentage of template-based reads (red dashed line). We can observe the mean template-based percentage among all characterized reads in each sample and the standard deviation reported by each tool: CRISPR-A, CRISPR-GA, and CRISPResso2. Differences between the three samples are significant (p-value <0.0001) performing an ANOVA and also in all cases when pairs of tools are compared (adjusted p-value <0.05) with TukeyHSD. B) Characterization of different modifications obtained by HDR. CRISPR-A and CRISPResso2 are used to characterize the result of 27 experiments with different modifications led by HDR: substitutions of one nucleotide by another, substitutions of 4 nucleotides for other 4 nucleotides, and insertions of 3 nucleotides. The modifications are found in 3 different targets. C) Characterization of substitutions done by BE. In this case, instead of looking for the overall percentage of editing we looked for the information of substitutions by position given by two different tools: CRISPR-A and CRISPResso2. There are 4 different targets and 3 replicates for each target. D) Characterization of PE substitutions. We have compared a total of 29 samples of PE editing of FANCF gene with templates with different PBS lengths. With CRISPResso2 analysis, the examples with a PBS shorter than 13 base pairs end up with error, and results are not obtained. E) Extended analysis of PE substitutions. 23 different samples edited in 5 different targets (HEK3, EMX1, FANCF, RNF2, and HEK4) are analyzed with CRISPR-A and CRISPResso2. In the plot on the left side, we can see the comparison of the reported PE percentage by each of the two tools. HEK3 edited samples have a higher PE reported by CRISPR-A than with CRISPResso2 (in orange). In the right part of the figure, we found the count of reads classified in each class. CRISPResso2 reports a high number of PE with modifications or reference with modifications due to a SNP (rs1572905; G >A).

CRISPR-A can be used to look for any kind of genetic mutation if a sequence with the change and its surrounding homology sequences are given. This allows reporting of HDR, PE, BE, or even microhomology-mediated end-joining (MMEJ) or NHEJ alleles of interest. Of the explored tools for indels characterization, CRISPResso2, CRISPR-GA, and CRISPR-A are the only tools capable of analyzing HDR events. These tools report values close to the expected percentage of template-based or objective modifications when simulated data is analyzed (Fig 3A). We added the same amount of reads with certain template-based modifications to the simulated data obtained with SimGE. Even if the amount of sequences with modifications is the same for each sample, the characteristic (size, position, and type) of each modification is different to have a comprehensive evaluation of the performance. The mean difference to the real percentage of sequences with the objective modification (1.09%) is 2-fold higher in CRISPResso2 than in CRISPR-A, while CRISPR-GA shows the closest mean distance to this value. Even so, there are some samples with a higher objective modification count, corresponding to indels that lead to the same modification as the template-based change. In general, CRISPR-A distribution is closer to the expected, centered on the percentage of template based added reads, and with low dispersion. CRISPResso2 shows a higher dispersed distribution, while CRISPR-GA tends to underestimate the objective modification percentage.

In addition to simulated data, we have analyzed targeted NGS data of HDR, BE, and PE experiments. The targeted NGS analyzed data of an HDR experiment [35] shows that CRISPResso2 is reporting slightly higher but significant HDR percentages than CRISPR-A in most of the cases (p<0.00005) (Fig 3B). However, what we can observe after exploring in detail 6 of the HDR analyzed samples (S3 Table) is that CRISPResso2 is classifying more reads as template-based than CRISPR-A, while CRISPR-A, in most of the cases, is classifying more reads as reads containing indels than CRISPResso2 does. We have sampled 383 reads to manually validate and understand better the different classifications between both tools. CRISPResso2 is reporting more reads as HDR edited than those found by manual inspection. In addition, it is reporting 45% less indels than in the ground truth, and 38.48% of the reads are classified as ambiguous, while there are just 8.36% of the reads unclassified when done manually. The counts of each class done by CRISPR-A, CRISPResso2, and manually can be seen in S4 Table. The samples with higher differences, almost 2-fold change, have a 42% of the reads classified as ambiguous by CRISPResso2, probably due to a substitution present in two thirds of the reads. Base edited samples [36] can also be analyzed as objective modifications by CRISPR-A if the percentage of a specific change in a particular position wants to be explored. In addition, CRISPR-A always searches substitutions along the whole amplicon sequence that can be used when all modifications at the quantification windows are of interest. CRISPResso2 and CRISPR-A give comparable results of substitution quantification in base editing experiments, using the default substitution search along the whole amplicon sequence done by CRISPR-A and indicating base editors as the editing tool to CRISPResso2 (Fig 3C). Prime editing (PE) is another case in which a template, the 3’ extension that is composed of Primer Binding Site (PBS) and reverse-template, is used to achieve an objective modification. We have analyzed FANCF gene modifications made with PE [35] and, even though results are similar when PBS is longer than 13 nucleotides, CRISPResso2 is not able to give results for PBS shorter than 13 nucleotides for this target, due to misleading alignment between 3’ extension and amplicon sequence (Fig 3D). We have extended these analyzes adding more samples from the same project and we observe that CRISPR-A gives higher editing percentages of template-based editing in several cases (Fig 3E). We have explored more in depth the cases where the difference was higher than 1.5%. This difference is explained by a high number of reads classified as reads with reference modifications or prime editing modifications by CRISPResso2. The sum of all these ambiguously classified reads is almost equal to the sum of the missing wild type, indels, or PE reads reported by CRISPR-A. All samples with these differences have the same target, and the undetermination is due to the fact that there is a SNP (rs1572905) in heterozygosity. We have manually classified a sub-sample of 383 reads from sample SRR10285861 (S5 Table). CRISPR-A correctly classifies 325 reads as wild type, 49 reads as template-based modified, and 4 reads as indels. It misleads in the classification of 5 reads: 2 indels classified as wild type and 3 indels that should be classified as WT. Without looking at the reads one by one, we will just be able to see that there is just one WT read less than expected, and 1 extra read classified as indel. In the case of CRISPResso2, we find 3 extra reads classified as modified wild type and one less as unmodified wild type. There is not a specific class for indels and there are 4 reads classified as ambiguous.

### Characterization of cell line dependent and independent editing outcomes with CRISPR-A

**Fig 4:**
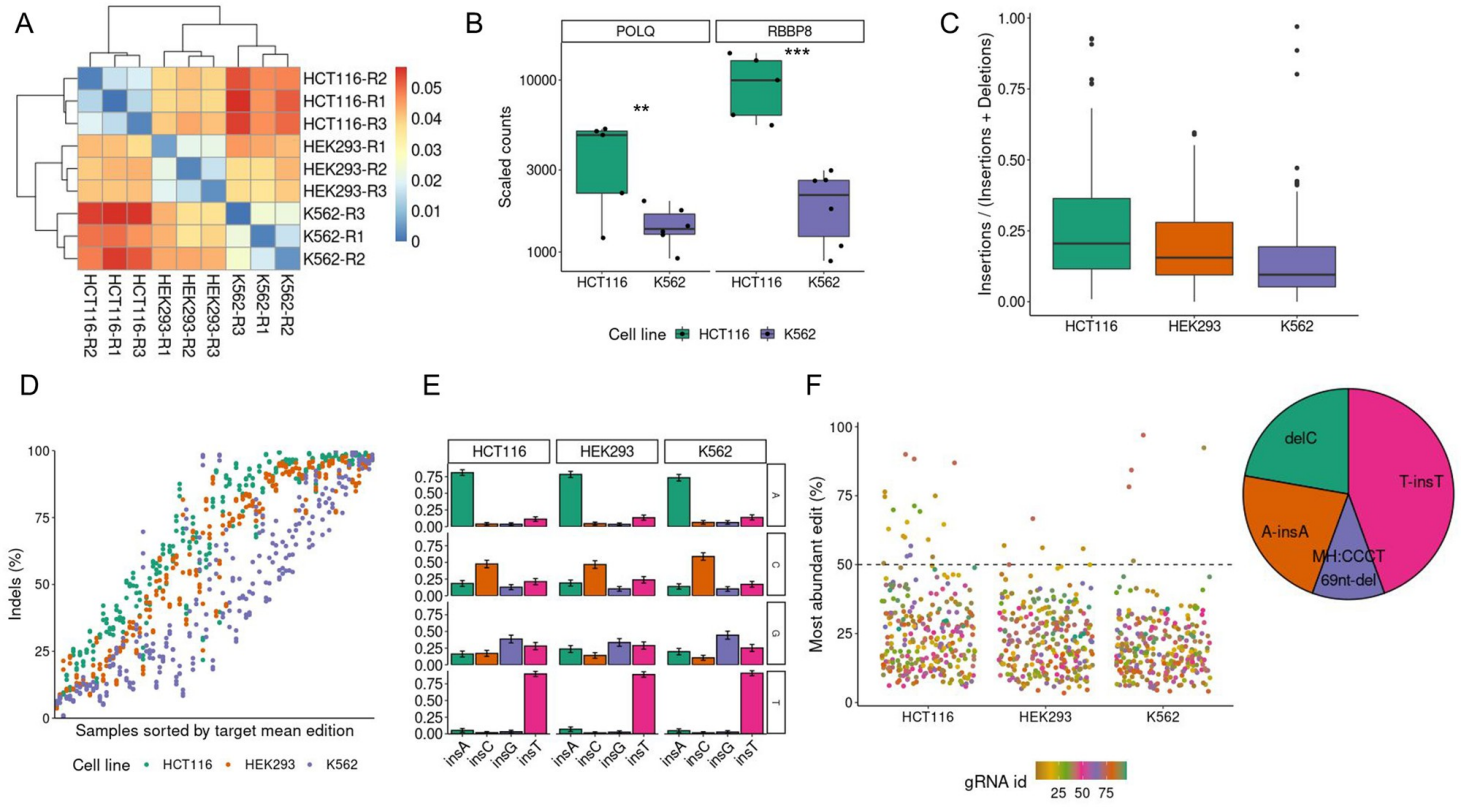
HCT116, HEK-293, and K562 edited in 96 different targets analyzed by CRISPR-A. A) MMEJ deletions ratio among all deletions. We have calculated the euclidean distance of the ratio of MMEJ deletions among all characterized deletions. The heatmap represents the hierarchical clustering of the mean between the euclidean distances calculated for each of the 96 different targets. The three replicates of each cell line are clustered together showing that the MMEJ ratio is a feature that depends on the cell line. B) Differential expression analysis of two genes associated with MMEJ in the two cell lines with higher differences in MMEJ ratio against NHEJ indel patterns in gene editing outcomes. Differences are significant (p-value < 0.05). C) Insertions against total edits. The proportion of insertions among all reported edits in three different cell lines. D) Percentage of editing. Indels among all characterized reads in 96 different targets with 3 replicates by each target in three different cell lines. E) Insertions considering the nucleotide upstream of the cut site. Percentages of inserted nucleotides in the function of the free nucleotide in three different cell lines. F) Variant diversity by samples. On the left is the percentage of the most abundant variant among all kinds of edits. In the right the distributions of variants from all the variants that are above 50% (dashed line in left plot), these are the variants with higher abundance.

Three different cell lines: HEK293, K562, and HCT116 were analyzed with the CRISPR-A analysis pipeline. The 96 samples, from these cell lines, are obtained from a public dataset BioProject PRJNA326019. The euclidean distance between the percentage of microhomology mediated end-joining deletions of samples with the same target was calculated and the mean of all these distances was used for the heatmap representation to reduce the information of the 96 different targets to a single one. After this, the replicates of each cell line cluster together (Fig 4A). In addition, we have done a differential expression analysis to explore the correlation of the DNA repair pathways (NHEJ and MMEJ) associated proteins of the two cell lines showing bigger differences with the gene editing landscape associated with these two same DNA repair pathways. The differential expression analysis has been done using data from BioProject PRJNA208620 and PRJNA304717. We have found that POLQ and RBBP8, also known as CtlP, are significantly more expressed in HCT116 than in K562, which are the cell lines with the major and minor ratios of MMEJ compared with NHEJ, respectively (Fig 4B). POLQ is essential for MMEJ in all mammalian species [37] and CtlP promotes MMEJ repair [38]. We also observe differences in the fraction of insertions among all indels, being HCT116 the cell line with the highest proportion of insertions among indels (Fig 4C). When we do an overall visualization of the percentage of indels observed in each sample, we also see different patterns among cell lines, with HCT116, a repair-deficient cell line, and HEK293 showing a higher percentage of editing than K562 (Fig 4D).

There are other CRISPR-based editing outcomes that instead of being characteristic of each cell line are common for all of them. This is the case of insertions of a single nucleotide at the cutting site, which shows a strong prevalence of insertion homology when there is thymine or adenine. This is not as frequent when the free nucleotide is a cytosine, and even less when it is guanine (Fig 4E). Another common feature is the diversity of variants by targets. We have explored the targets that have an editing outcome that represents more than half of all different edits in a certain sample. Of the 96 gRNAs, there are 9 different gRNAs which lead to outcomes with low diversity of editing genotypes. We can see certain strong patterns on these targets: free thymine or adenine at the 3’ nucleotide upstream of the cutting site that lead to insertions of the same nucleotide, a free cytosine at the same place that leads to its loss, and strong mirco-homology patterns that lead to a long deletion (Fig 4F).

### Three different approaches to increase precise editing outcomes counts determination

We have implemented three different approaches to increase edits quantification accuracy: (i) synthetic molecules of known size and quantity (spike-in controls) to model size biases, (ii) UMIs to remove PCR duplicates, and (iii) an empirical model based on mock samples to denoise the treated ones.

**Fig 5:**
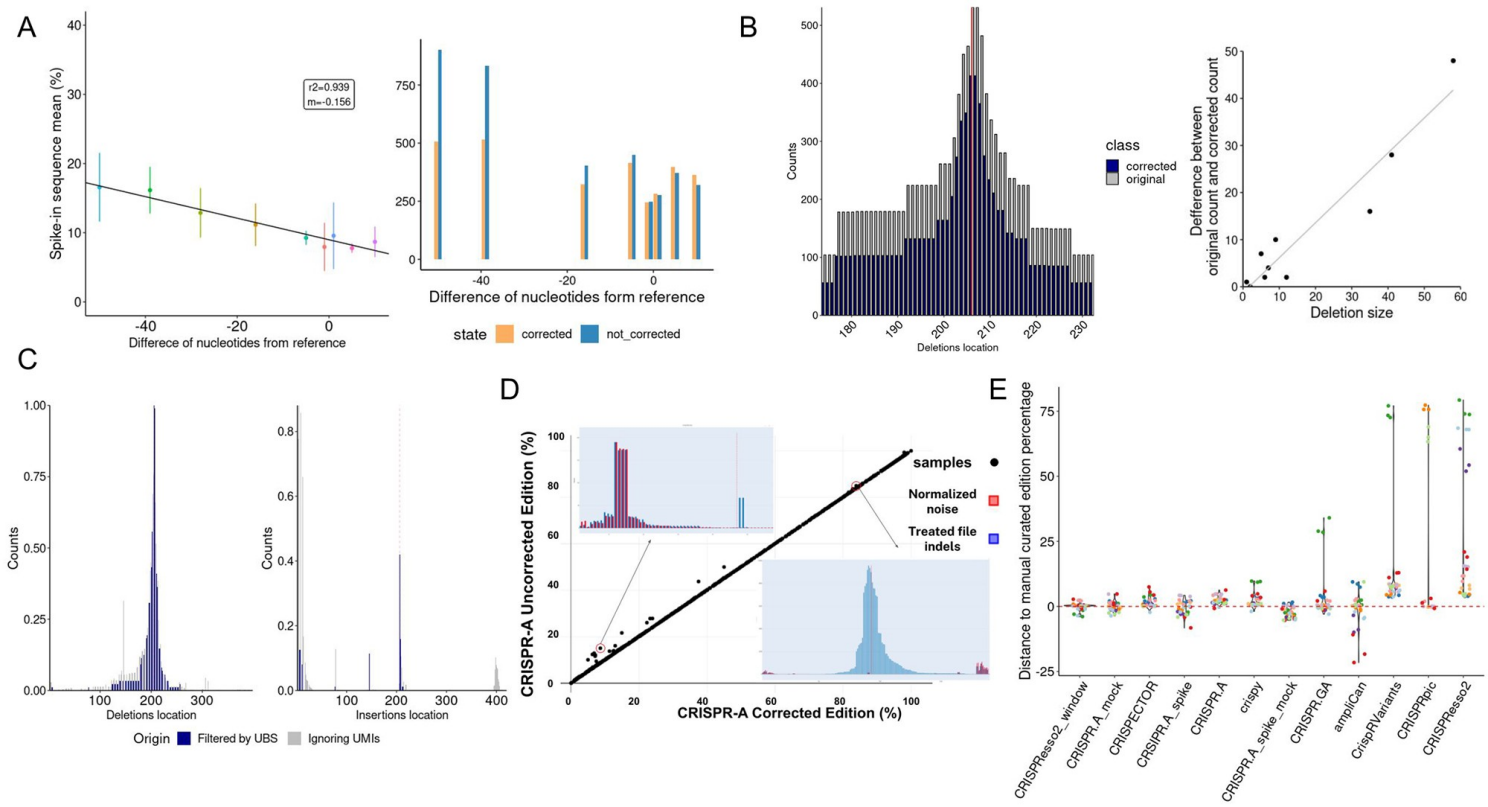
Enhanced precision with spikes, UMIs, and mock characterization. A) Spikes count in the Illumina experiment. Count of spike-in synthetic sequences with different deletion sizes. From each spike, the same number of molecules were added to the edited samples. On the left, linear regression of spike-in sequences mean percentages among all spike-in sequences at 30 cycles of amplification and a low number of molecules. At the right, count of spikes in the original sample and after correction by spikes. B) Size bias correction using the spike-in model. On the left, edited sample deletions distribution by position corrected by spikes (blue) against the original distribution (gray). At the bottom right, count difference between original and corrected in function for deletion size of sample deletions distribution shown at left. C) Noise reduction by UMIs cluster filtering. Standardized distribution of deletions (left) and insertions (right) without taking into account UMIs (gray) and after clustering by UMIs with a minimal identity of 0.95 and filtering by UMI bin size (UBS) >50 and <130 (blue) in Lama2 target. The Red dashed line corresponds to the cut site position. D) Mock-based noise correction. Samples with less percentage of editing tend to have a higher correction since the noise represents a higher proportion of the indel reads. The two plots comparing treated and mock files show that this subtraction is always specific, regardless of the editing percentage. E) Difference between the editing percentage reported by 8 tools and the 4 options of CRISPR-A and the manually curated percentage of 30 samples (10 different targets and 3 replicates for each).

Synthetic molecules with known deletions and insertions of different sizes in relation to the reference sequence, called spike-in controls, were analyzed together with edited C2C12 cells genomes. The differences in the final count of the spikes increased together with the number of Polymerase Chain Reaction (PCR) cycles, and the number of initial molecules is not as relevant as the number of cycles (S9 Fig). After observing the linear correlation between the size of the spikes and their relative count, we have fitted a linear model to transform the indels count depending on its difference in relation to the reference amplicon (Fig 5A). When we compare the original count with the updated counts after size-based correction, we see that the distribution of indels is flatter (Fig 5B).

Using UMIs we can cluster several reads that come from the same original molecule and see if the indels and substitutions found are errors or are real changes produced before the sequencing process. We have used two different amplicons (TRAC and Lama2) to explore the percentage of reported indels equal to the consensus of the cluster. The explored clusters were generated using different minimum allowed sequence identities. The identity is the number of alignment columns containing matched residues between the length of columns after alignment; we explored 80, 85, 90, 95, and 99 minimum allowed identity within the clusters. We saw that smaller clusters tend to show less percentage of reads supporting the consensus sequence, while with clusters that have an identity lower than 90% the dispersion is maintained since bigger clusters are achieved (S10A Fig).

One example, where the effect of using UMIs, clustered by an identity of 95, to filter data can be seen is shown in Fig 5C. The sequenced target has a high sequencing complexity that leads to imprecise sequencing. In gray, we can see indels normalized counts before applying UMIs clustering and cluster filtering. After filtering by UMI bin size (UBS) higher than 50, removing in this way the sequences with the same origin but contained in several clusters, and by UBS lower than 130, removing sequences with different origins in the same cluster, we see that noisy positions are filtered. Removal of noise using the same approach can also be seen using other targets as TRAC (S10B Fig).

Lastly, CRISPR-A uses an empirical approach to address the misled classification of errors as editing events, requiring a negative control (mock file) to denoise treated samples (Fig 5D). This step of the analysis is optional and flexible, allowing combinations of samples to be corrected with the same or different mock files. The model compares the indels occurring in the mock and in the treated file separated by indel type (insertion or deletion), size in bp, and position relative to the cut site to avoid biases. Significant differences in these features were observed between the indels in the mock and in the treated files present in a data set [27] with three different cell lines (HEK293, K562, and HCT116) that had negative controls for the three biological replicates of each gRNA (S11A, S11B and S11C Fig).

Finally, we have compared the different versions of CRISPR-A (using or not a negative control, and correcting or not size biases by spike-in control) and all other tools explored in the benchmarking with a manually curated data set (Fig 5E). The use of a negative control sample when running CRISPR-A allows obtaining results with a lower difference to the manually curated values. CRISPResso2 has a high dependency on the quantification window determined by the cutting site. When CRISPResso2 is used with a quantification window, it is on the top, while without specifying this window, it is the tool performing worse. CRISPRpic is the worst performing tool. It only accurately characterized 12 of the 30 analyzed samples and is far from the correct editing percentage in other 6 samples. In the other 12 samples, CRISPRpic does not give back any result. CRISPR-GA, cris.py, CripRVariants, and CRISPECTOR show, in general, values that are higher than the manual curated value. AmpliCan has outliers on both sides. Similar results can be observed when instead of a manually curated data set, a sub-sampling humanly validated data set is used as ground truth (S12 Fig). Since the reads characterized by humans can be classified as true positive (indels that are edits), true negative (wild type classified as wild type), false positive (indels that are errors) and false negative (edits classified as wild type), we have calculated the accuracy of CRISPR-A classifying these more than 3,500 reads from edited samples. CRISPR-A accuracy is 0.95, when edits discarded are also counted as classified reads.

### CRISPR-A empirical model removes more noise than other approaches

The fact that a model subtracts more or fewer indels does not necessarily mean that it removes more noise, since it may be masking bona fide indels. Therefore, to establish a framework on which we can compare our model we looked for how many indels with the same characteristics (class, position, and size), we could find at the same time in the mock and treated samples from the data set [26] of 96 different targets in three different cell lines that a negative control for each cell line and target (S13A Fig). What we observed is that the representativeness of the mock files, although quite high near the cut site, is not total in the rest of the sequence, i.e, that there are indels introduced by the noise in the mock files that do not have an equivalent in the treated ones. Moreover, when calculating the proportion of treated file indels represented in their mock files (S13B Fig) we found that it is much more likely that the reported indels far from the cutting site are noise since they are proportionally more represented. Knowing this, we calculated the subtraction efficiency of our model (S13C Fig), obtaining that, of the indels represented, those that are in higher proportion (far from the cut site) are almost completely subtracted while those that are less proportionally represented (close to the cut site) are less subtracted. It is also observed that after normalizing by aligned read depth, these characteristics improve (S13D Fig) suggesting that normalization and proportional subtraction generate an efficient and sensitive model by minimizing the possibility of masking bona fide edits that are represented by chance.

After characterizing the efficiency and sensitivity of our model, we could do benchmarking against other denoise approaches. This would let us know if this novel approach improved somehow the state-of-the-art. Among all the tools presented in the introduction, only ampliCan and CRISPECTOR apply some kind of noise correction model. To benchmark the CRISPR-A model, we compared its performance with the previously characterized data set.

AmpliCan evaluates position by position the amount of noise, and if it exceeds a certain threshold, it considers all indels in that position as noisy, regardless of the class. Meanwhile, CRISPECTOR uses a Bayesian model with configurable priors that evaluate positions as noisy or not, stratifying by indel type, and position.

The results were surprising, as the subtraction applied by ampliCan had zero effect on the reported editing percentage. In the case of CRISPECTOR, a correction, which is a change in the editing percentage using a mock to denoise compared without using it, was observed, although very modest, since only about 10% of the files underwent a correction. For CRISPR-A this percentage went up to 82% (S14A Fig).

To better understand where these differences may be occurring, we explored the correction of some of these samples. It should be noted that this comparison was difficult to evaluate as CRISPECTOR, although providing extensive details on the statistics and information about the indels, is not possible to track the reads along their pipeline, thus we cannot know which have been corrected and which have not.

For instance, sample SRR3700075 has a repetitive sequence region at the beginning of the sequence (CTG CTG CTG CTG CAG CAG CAG) that produces a peak of indels, probably due to noise. This peak partially masks the peak next to the cut site. CRISPR-A effectively corrects this region since it is equally represented in the mock, while CRISPECTOR shows a correction close to zero.

An opposite case is SRR3696877, where we observe a greater correction of CRISPECTOR than the one applied by CRISPR-A. As can be seen in S14B Fig, besides a few noisy insertions at the extremes, there’s not much subtraction going on. Some deletions next to the cut site were tagged as noisy, but not subtracted. It is difficult to assess if those deletions correspond to the extra correction that CRISPECTOR performed, but CRISPR-A limited this subtraction since, in the mock file on those positions, the observed normalized frequency was lower than the treated, avoiding masking.

Finally, it is worth mentioning that there are samples that CRISPECTOR does not analyze due to an imbalance between mock and treated samples. Since we saw no correlation between depth reads and the representativity (S15 Fig), we concluded that the subtraction can still be performed. That’s why CRISPR-A does denoise the samples while notifying the user that the depth is lower than the arbitrary threshold of 300 reads, as in SRR3698365.

## Discussion

NGS is the method that enables the identification of all different outcomes led by genome editing tools. There are different online and command line available tools to decipher the percentage of edits achieved in genome editing experiments. Even so, most of these tools do not retrieve all possible kinds of editing events and are not flexible enough to cover the whole diversity of genome editing tools. Moreover, none of them include simulation to help in the design or analysis performance evaluation. Furthermore, alignment, amplification, and sequencing errors have not been previously taken into account systematically to achieve a precise estimation of CRISPR-based experiments results. Neither spike-in controls or Uni-Molecular Clustering had been applied in this field to correct these errors.

Here we described CRISPR-A, a tool that can be used to simulate gene editing data as well as analyze the efficiency and outcomes profile of any kind of CRISPR-based experiment. Our tool has achieved a higher performance to those of current applications, with additional versatility, and with a dynamic oriented reporting that enables its use by a wide scope of users. In CRISPR-A analysis, there are no biases due to assumptions related to the performance of the used technology since the analyzes are agnostic to the used gene editing tool and the denoising process is done using an empirical model. This makes the tool suitable for the fast development of the genome editing field. The novelty of the applied methods such as the use of UMIs and spike-in controls for quantification correction for an enhancement in precision is unique among the current CRISPR-based analysis tools and it may facilitate its use in situations where accuracy is paramount (i.e: clinical). We have also improved the use of mock samples in the correction of confounding errors. Another unique functionality of CRISPR-A is the detection of the reference amplicon sequence from a reference genome. This functionality is useful for different things: first, to save time avoiding the need of looking for the sequence of each amplicon and adding it as input, second, it can help to detect unspecific amplification, since all amplicons of the same genome can be retrieved and inspected from the discovery amplicon process, and third, we have already shown one example where this avoids getting incorrect results since the use of the whole reference amplicon obtained with CRISPR-A amplicon sequence discovery function leads to CRISPR-A showing a perfect edition profile.

We have coupled the analysis pipeline with a simulator that has been useful to benchmark gene editing tools. The probability distributions of the algorithm were fitted with 4 different cell lines to capture the peculiarities of each one of them, which makes it more generalizable. After exploring different distance metrics, we determined that Jensen-Shannon divergence is the best measure to compare these probability distributions similarity. We succeeded, since the distance within samples with the same target, simulated and real, had a lower distance than between different sample targets. In addition, we are not able to separate and distinguish samples that are replicates, since its simulations will come from the same input. As expected, we find lower distances between these two real samples as well as between both of them and their simulation compared to different samples (real or simulated) with a different target. On the side of the analysis, alignment matrix penalty values were tuned to obtain a better edit calling. When current tools were benchmarked with simulated data, even without using spikes or mock correction, only CRISPResso2 approached CRISPR-A accuracy. While with real data, these two tools can only be compared if CRISPResso2 uses a filtering window based on the cut site inferred from the protospacer sequence. This implies a poorer editing report of CRISPResso2, compared with CRISPR-A, in the samples where the cleavage site is slided. Even so, window filtering can sometimes be convenient to remove noise, for that reason we have implemented other approaches with this aim like an empirical model based on mock samples that is useful to eliminate the noise produced from multiple sources, such as sequencing errors, misalignments, natural variants, etc. CRISPR-A with mock correction is the most accurate tool. In addition, after the analysis CRISPR-A allows filtering by windows interactively; this is helpful, when a mock sample is not available, to remove alignment errors in the primer binding site (PBS) that can be observed in the extremes of the amplicon. Moreover, lately we implemented an extra process when there is no mock sample to filter repetitive regions, that tend to be noisy, and indels at PBS. This makes CRISPR-A simplest version really accurate. In addition to double strand break repair characterization, CRISPR-A looks for any kind of genetic mutations when a sequence with the change and its surrounding homology sequences are given. Even when an objective modification is not given, CRISPR-A reports all found substitutions along the amplicon. Successful results with different kinds of data and approaches were achieved. One limitation of the agnostic search of substitutions is that the report is not given separated by substitutions in the same read, it just gives an overall percentage by position. Meanwhile, the obtained results of objective modifications show an improved performance compared with current tools. The main difference between CRISPR-A and CRISPResso2 when reporting PE events is that CRISPResso2 is not reporting indels in samples with more than 1% of indels. Reporting indels when analyzing Prime Editing data is really relevant since undesired indels are a common outcome of Prime Editing [39]. Similarly happens with HDR, where we have verified that CRISPResso2 classifies more reads as HDR than there are and almost half of the indels are not detected by this tool.

CRISPR-A has enough precision to find relevant features and outcomes in different cell lines. We have used CRISPR-A to characterize gene editing outcomes in three relevant cell lines (HEK293, HCT116 and K562). We have found certain patterns associated with low diversity outcomes: free thymine or adenine at the 3’ nucleotide upstream of the cut site that leads to insertions of the same nucleotide, a free cytosine at the same place that leads to its loss, and strong micro-homology patterns that lead to long deletions. These kinds of patterns that lead to a certain repair resolution with low variability are of high interest when a certain consequence of the cut has to be achieved. For this reason, CRISPR-A analysis report shows the edited variants with higher prevalence in analyzed samples and its frequency.

Precision has been enhanced in CRISPR-A through three different approaches. The observed inverse correlation between amplicon size and over amplification, has been used to correct size biases and obtain flatter distributions of indels. We also removed indels in noisy positions when the consensus of clusterized sequences by UMI are used after filtering by UBS. Finally, an empirical model based on mock samples has been implemented to denoise the treated ones exceeding the tools that implement similar methods.

While CRISPR-A provides a high performance platform for gene editing analysis, it will continue evolving to incorporate future improvements. There is still room to explore UMIs and spike-in correction methods more in-depth. At that moment, the analysis pipeline that clusters UMIs is independent of the nature of the sequencing technology used. In the case of spike-in controls, a more complex model simulating PCR amplification could be explored and developed. Another area of improvement is the noise subtraction model since it requires a negative control which is not always available. To solve this, a mock-independent model that takes into account the trends that we have observed in noise, together with generally described noise signatures such as homopolymeric regions or high/low GC content [40], could be implemented to follow CRISPR-A minimal assumption philosophy.

## Methods

### Simulations algorithm development

SimGE is built taking into account the different layers of classes and their proportions. The proportion of edited and not edited sequences can be determined by two different sgRNA efficiency predictors, Moreno-Mateos [41] and Doench 2016 [42] scores, which give the most reliable on-target activity prediction [43]. Both models give a group of weights that are assigned as descriptors of the gRNA sequence in order to define the efficiency as a value that falls between 0 and 1, being 1 the most efficient gRNA. Depending on the experimental design, one model or another suit better the data: for guides expressed in cells from exogenous promoters, like U6, Doench 2016 scores are recommended, but for guides transcribed in vitro from T7 RNA polymerase promoter, using Moreno-Mateos scores is a better option.

Deletions and insertions location distributions are determined by the fitted distributions. The same happens with deletions sizes. Insertions, by default, have a size of 1 nucleotide. The inserted nucleotide is determined by a conditioned probability, taking into account the nucleotide upstream to the cut site, since insertion homology is a feature with high prevalence [44].

Furthermore, the sequence context is more relevant in microhomology mediated end-joining (MMEJ) deletions than in non-homologous end-joining (NHEJ) deletions, since for MMEJ deletion an homological pattern on both sides of the cut site is required. For the NHEJ ones, the specific nucleotides are removed based on the probability density function of the sizes and the probability distribution of the positions relative to the cut site. To determine the proportion of each MMEJ deletion and NHEJ:MMEJ ratio, we calculate the microhomology (MH) score based on the MH pattern and the length of the deletion, similarly as done by Bae et al. [45].

### Simulations evaluation

With the purpose of being able to compare the different sample sizes and the positions of the indels, we needed to define a distance metric. When clustering the observations into groups, we computed the distance between each pair of observations (S6 Table), giving an idea about the dissimilarity among the observations. The explored distance metrics were: Euclidean mean, Euclidean median, Kullback-leibler, and Jensen. Euclidean distance is defined as the length of a line segment between two points in Euclidean space. If columns have values with differing scales, like the indels for different samples, it is common to normalize or standardize the numerical values across all columns prior to calculating the Euclidean distance. Otherwise, columns that have large values will dominate the distance measure. Euclidean distance is calculated as the square root of the sum of the squared differences between the two. Using the median instead of the mean and a more robust dissimilarity metric is much less sensitive to outliers. Median and quantiles are not suitable to describe the distribution if we have few categories, being the standard deviation a better fit. Previously, mutual information, entropy, and Kullback-Leibler (KL) distance have been used to study different parameters [46]. Hu and Hong use KL with the goal of studying the changes in isoenzyme expression, being the entropy, a measure to quantify complexity, predictability, and progression patterns. Jensen-Shannon (JS) divergence and distance have been used to explore probability distributions, being considered the symmetric and smoother version of KL divergence. Using the Jensen-Shannon and Kullback-Leibler metrics, we aimed to analyze both size and position of indels between samples.

We used five-fold cross-validation to train SimGE. We split the T cell data [26], which includes 1.521 unique cut sites, into five-folds and trained SimGE on four of the five folds (S3B Fig). The same process was performed for the other three different cell lines, including 96 unique cut sites and three replicates [27]. In order to obtain the different distributions, the T cell data, including 1.521 unique cut sites, was split into different datasets based on the different classes: deletions, insertions, and substitutions. For each of these classes, giving as input the datasets with only that class, we obtained the distribution for size and then for the position of indels. The same was done for the other three cell lines: K562, HEK293, and HCT116, which included 96 unique cut sites, with three replicates each. The whole datasets (with 1521 and 96 unique cut sites) were split into five-folds (4 for training and one for testing) and validation, in order to train and validate the simulator. Using the parameters obtained during the training-test iterations (the average value of the 5 iterations), we generate simulated sequences with the same target and gRNA as the samples that are assigned to the test subset to calculate the Jensen-Shannon (JS) divergence between the simulated and real samples of that subset. Finally, the same was performed for validation. The input for the distance calculations were the generated simulated subset and its real equivalent (same target and gRNA) distributions of the classes.

### CRISPR-A gene editing analysis pipeline

In case of working with pooled samples, the demultiplexing of the samples has to be done before running CRISPR-A analysis pipeline using the proper software in function of the sequencing platform used. The resulting FASTQ files are the main input of the pipeline. When paired-end reads are used, the merge of the reads is done with PEAR [47]. After that, FastQC [48] and Cutadapt [49] are used for the detection and removal of adapters. Then, quality filtering is done with fastq_quality_trimmer from fastx-toolkit http://hannonlab.cshl.edu/fastx_toolkit with a quality threshold of 20 and a minimum length of 80. An adapted version of extract_umis.py script from pipeline_umi_amplicon pipeline (distributed by ONT https://github.com/nanoporetech/pipeline-umi-amplicon) is used to get UMI sequences from the reads, when the three PCRs experimental protocol is applied. Then vsearch [50] is used to cluster UMI sequences. UMIs are polished using minimap2 [33] and racon [51] and consensus sequences are obtained using minialign (https://github.com/ocxtal/minialign) and medaka (https://github.com/nanoporetech/medaka).

If instead of the amplicon reference sequence the genome reference is used, reads are aligned using BWA-MEM [30]. After that, samtools [52], bedtools [53], and custom scripts are used to get the reference amplicon sequence. This process is based on alignment coverage and length of the region with aligned reads above it. Before aligning reads against the amplicon reference sequence with minimap2 [33] using the following parameters: -A 29 -B 17 -O 25 -E 2, the amplicon reference sequence is placed in the same orientation as the gRNA to get standardized reports for all analyzed samples. Instead of using minimap, other aligners such as BWA-MEM [30] and Bowtie2 [31] can be used.

When there is not a mock sample to do the noise subtraction, there is a script to define repetitive regions in the amplicon reference sequence. All K-mers of length 1 to 6 with step one and starting from all possible beginnings (as many possible starts as K) are retrieved if they are repeated at least 4 times. These intervals of repeated regions are ignored in the variant calling step if they are not above the cut site. In addition, when the cut site is at least 15 nts above the start or end of the reference sequence, these 15nts are also ignored. In case that the cut site is closer to the extremes of the sequence, just the 2 first or last nucleotides are ignored.

Variant calling or edit calling is done based on the cigar sequence and using custom scripts. Deletion’s position and extension are determined by being aware of the nature of the cut and repair mechanisms. For that reason, the deletions considered are not truncated and when different possibilities arise, the expression of deletions corresponding to those that are above the cut site are chosen. Substitutions by positions are also reported using pileup from samtools [52], and in case of the existence of an objective modification, we look for it by aligning the reference against the modified reference and counting the cigars with the same pattern. When there are no deletions or indels in the cigar, substitutions are searched after doing pairwise alignment to get the position, size, and nature of the substitution.

Also in the variant calling process, spike experimental data is optionally used to correct size biases of deletions and insertions. The proportion of reads is removed (for deletions) or added (for insertions) taking into account the linear regression observed when spikes of different sizes and at the same initial amount were counted. The transformation is done using the following formula, where m is the slope of the linear regression and n is the origin:

$$\begin{array}{c} OriginalCount=ObservedCount+\frac{ObservedCount\;*\;m*Difference}{Difference\;*\;m+n} \end{array}$$ (1)

If the amplified region of the treated samples is also amplified without treatment (mock), this negative control is used to remove noise.

A linear regression model was fitted to obtain the parameters of Eq 1 using spike-in controls experimental data (original count, observed count and size of the change in the synthetic molecules). We have used the lm function from R. Parameter m in Eq 1 is equivalent to the obtained coefficient estimate of x which was 0.156 and n is the intercept (n = 10).

### CRISPR-A noise subtraction pipeline

To apply the subtraction, the mock file is analyzed in an agnostic manner, following the same path as treated files, in an independent manner. The idea behind it is that the complexity of sources that can lead to technical noise should be represented on a control that has undergone the same process with the same sequence. Thus, after performing the edit calling, the mock samples are paired with their respective treated samples.

The first step is a recovery of truncated events, in which reads classified as truncated in the treated file are compared against the mock. This allows to recover real edits that could not be classified due to a noise event occurring in the same read. Right after aligned read depth normalization occurs, we obtain a normalization ratio using the following equation:

$$\begin{array}{c} NormalizationRatio=\frac{TreatedAlignedReads}{MockAlignedReads} \end{array}$$ (2)

This ratio is then used to multiply the number of noise events found on the mock, gathered by length position and indel class, to avoid biases. Note that this also occurs when there are more aligned reads on the mock than on the indels, but instead of multiplying, it divides the indels to avoid over subtraction. After normalization, a simple subtraction is performed based on the number of indels observed in the control, being subtracted from the treated file only those indels that match in position, length, and edit class (insertion or deletion). After this process, if there are still reads in the treated file that meet these conditions but that has not been subtracted because they occur proportionally more frequently in the treated sample than in the negative control sample, they are tagged as noisy reads differentiating them regarding their relative frequency, i.e, how big was the difference in counts between them. The reads subtracted are then classified as wild type and, for that, indels and wild type percentages are recalculated. All these processes are based on R 3.6. and included in the nextflow pipeline.

### Alignment and gene editing analysis tools benchmarking with simulated and real data

SimGE was used to simulate more than one hundred sequenced edited targets. The coordinates of the sequences were randomly selected from the human genome (hg38) with bedtools. Samtools was used to get the fasta sequences and a custom script was used to select a SpCas9 possible cut site at least 35 nucleotides away from both extremes of the sequence. After converting the FASTAs with the simulated edits into FASTQs with SeqIO from Biopython, reads were aligned against reference amplicon sequences using BWA-MEM, BLAT, minimap2, and pairwise alignments to compare their performance. The accuracy of the alignment of each sample by each aligner was computed dividing the number of reads correctly classified by the total amount of reads. Pairwise alignment was the slowest option and minimap2, was the second best aligner. We explored the performance of minimap2 tuning the matching score, mismatching penalty, gap open penalty, and gap extension penalty. To achieve it, we have done a random sampling of parameter combinations and we have analyzed the alignments to calculate again the accuracy for all samples. From PCA analysis we can see the relevance and the space of combinations that move us to more accurate results (Fig 2B). Other PCA representations can be found in S16 Fig.

These simulated data sets were also analyzed with CRISPR-A after updating the alignment parameters and other popular tools (CRIPRResso2, CRISPR-GA, crispr.py, crispRVariants, and CRIPRpic). CRISPREsso2 (version 2.2.5) was run with docker and using default parameters. CRISPR-GA was run through its web application. R 3.6.0 was used to analyze data with CrispRVariants (version 1.14.0). In this case, we have just used the function indelPercent to get the percentage from reads aligned with BWA-MEM as in their usage examples. We have used an environment of python 2.7 to run crispr.py v2 and CRISPRpic.

We also analyzed the primary T cells data set [BioProject PRJNA486372] [26] using the same parameters for each tool. In the case of cris.py we have pre-merged the reads since it does not work with separated pair-end reads.

### Template-based and substitutions count with CRISPR-A, CRISPR-GA, and CRISPResso2

We have used the previously described simulated data set adding 1100 reads with different template-based modifications to compare the performance of these two tools. We have used a custom python script to generate templates and modified reads with diverse objective modifications including substitutions, insertions, deletions, and delins with different distances to the cut site.

CRISPR-A template-based edits search is based on CIGAR as well as indels search. Although, in this particular case, the user wants to quantify a certain change done with HDR, PE, BE, or any other CRISPR-based technology capable of generating precise edits. For that reason, the quantification is done using the given template and the other counters are updated taking into account the nature of the change. First, the reference is modified using the template that should be shaped by the modification and homology arms in both sites of the change. Then, the new reference with the modification incorporated is aligned against the template with the same algorithm and parameters used to align the sample reads.

Finally, when there are indels in the template, the reads with the same CIGAR are considered as template-based edits. When the template contains substitutions, these are collected taking into account the location and nature of the substitutions.

CRISPR-GA web application was used to quantify the template based simulated data sets using the same templates with different homology arms sizes as used with CRISPR-A, while in the case of CRISPResso2 what we have used is the amplicon reference with the modification integrated with the template. To quantify the template-based reads, as well as base edited reads, with CRISPResso2 we used the same docker as for indels quantification. In the case of prime editing analysis, we get an error that makes us move to the more recent version of CRISPResso2 (2.2.6) that only could be installed with conda in a python3 environment.

### Manual curation of 30 edited samples

We have classified aligned reads as wt or indels with human assessment of 30 different samples from HCT116 edited cell lines [BioProject PRJNA326019]. The 10 targets selected were those with higher editing differences between analyzing using or not a mock for correction. First, sequences with no indels, after alignment of sequencing reads against amplicon reference with minimap2, were classified as wild type. The counts of aligned reads with indels were recalculated using a size bias correction model based on spike-in controls empirical data (Eq 1). Reads with one indel were classified as indels from editing or as wild type, when assuming that the indel is noise, taking into account its length and position respective to the cutting site. First, those indels that were not above the cut site, were classified as wild type reads assuming that the indels were noisy. Then, those indels that were not above the cleavage site were manually inspected to decide if their more probable origin was noise or editing. In the case that the cut site could be slided by an adjacent PAM sequence or when some nucleotides can be moved from one side of the alignment to the other side to make the indel go through the cleavage site, the reads were classified as edits. A similar approach was followed for reads with more than one indel. In this case, the main question was if at least one of the indels was probably caused by the editing machinery.

### Human validation of 10 edited samples

We have used the same 10 targets as in the manual curation described above, but instead of the three replicates, just one sample for each target: SRR3700075, SRR3699982, SRR3699817, SRR3700021, SRR3699916, SRR3699952, SRR3699955, SRR3699901, SRR3700042 and SRR3699898. In addition, we have included one of the samples edited with prime editing; sample SRR10285861, and another one for HDR (SRR10286522). For each sample we have calculated the minimum sample size to get a confidence level of 95%, a margin error of 5% and a population proportion of 50%. In all cases, the sampling size is around 300. We have used the random package from python to make the sampling of the reads from the FASTQ files downloaded from SRA. The sampled reads have been saved in excel sheets representing the numbers of the reference positions in the first row (one cell per value), the reference in the second row (one nucleotide per row), and the aligned reads in the following rows. In the case of reads, each cell can contain a single nucleotide, many nucleotides when insertions are detected or a hyphen if there is a deletion. In addition, the first column was a checkbox to show conformity or nonconformity with the representation of the read alignment and the nomenclature attributed by CRISPR-A, which describes the nature of the indel. Moreover, there is a second column to classify the event as edit, wt, error, error+edit, edit+error and template. This last class was just used for the prime editing sample. 15 different humans have participated in the examination of more than 3,000 reads. All of them had the same PDF with the instructions to proceed in the examination of around 100 of reads to validate. With these human validated events, we have calculated CRISPR-A accuracy, dividend the number of events classified correctly by the total number of classificable events, and we have obtained a percentage of edition dividing the edition events by the wild type events classified by the 15 humans.

### Exploration of trends in noise

The feature distributions were obtained after comparing the indels detected in 288 control samples, with the ones detected in 864 samples from 3 different cell lines. The comparisons were done by cell line and biological replicate, being this 96 vs 96 samples to not incur on size bias.

### Subtraction performance assessment

All the samples had equal sized amplicons that had their cut site at position 47, allowing stacking and position assessment. To evaluate the performance of the model, we define the parameters of representativity (Eq 3) and efficiency (Eq 4). The formal definitions of these parameters are stated below. All these parameters are calculated by position.

$$\begin{array}{c} Representativity\%=\frac{Indels\in TreatedMatchingClassesOnMock}{TotalTreatedIndels}*100 \end{array}$$ (3)

$$\begin{array}{c} SubtractionEfficiency\%=\frac{IndelsSustracted}{TotalRepresentedIndels} \end{array}$$ (4)

To calculate these, we applied the subtraction algorithm without normalizing, normalizing, and inverse. In other words, comparing the edited file with the normalized mock and not the other way around. To consult the code, read “Data and materials availability”.

### Noise subtraction comparison with ampliCan and CRISPECTOR

ampliCan required the primers used to amplify the amplicon. Since in the data from Van Overbeek et al. paper [26] [BioProject PRJNA326019] they were not specified, we assumed 15 nucleotides from the start and end of the given amplicon sequences. Some of these sequences were substituted by their reversed complement to allow matching of the reads for ampliCan characterization. Otherwise, the tool does not report edits. Finally, to perform the comparison, the column “Control” from its config file was set to FALSE as it is optional.

As for CRISPECTOR, since it requires a mock file to perform on-target analysis, synthetic mock files were generated with the same number of reads reported by this tool for their respective real mock file. In these files, for each sample, all reads correspond to a sequence identical to the provided amplicon so they can be considered mocks free of noise events. From the different resulting parameters, we used the number of edited reads as a reference for both tools.

### HEK293, HCT116, and K563 data analysis

96 targets from 3 different cell lines with three biological replicates per target, with genomic extraction, 48 hours after transfection were analyzed with CRISPR-A analysis pipeline [26] [BioProject PRJNA326019]. CRISPR-A returns a table with all reads characterized with indels and descriptors of the indels characteristics: kind of modification, position, length, read identifier, micro-homology patterns, nucleotides before the insertion, inserted nucleotides, and nucleotides after the insertion. The content of these tables has been processed with custom R scripts to visualize and explore the results.

RNAseq differential expression analysis of samples from BioProject PRJNA208620 and PRJNA304717 was performed using nf-core/rnaseq pipeline [54].

### Cloning and plasmids

pDNA-Lama2-gR271 plasmid was performed by Golden Gate assembly using BsaI enzyme and standard methods, by fusing pDNA-BsaI plasmid (vector with Ampicillin resistant gene, the origin of replication derived from the puc19 plasmid and a cloning cassette with BsaI cloning sites) and the Lama2–271-BsaI amplified from C2C12 gDNA.

Different mutations were introduced into pDNA-Lama2-gR271 by site directed mutagenesis following Q5 Hot Start High-Fidelity 2X Master Mix (NEB) to employ as synthetic molecules of known size and quantity (spike-in controls) to model size biases. Primers were designed to achieve the following indels: 50 nt deletion from 243 nucleotides away from the first amplified nucleotide for targeted sequencing (50_del), deletion of 5 nucleotides from position 277 (5_del), deletion of 1 nucleotide from position 281 (1_del), deletion of 28 nucleotides from position 243 (28_del), deletion of 39 nucleotides from position 243 (39_del), 1 nucleotide insertion at position 281 (1_ins), 5 nucleotides insertion at position 281 (5_ins), and 10 nucleotides insertion at position 281 (10_ins).

### Cell culture, transfection, and electroporation

C2C12 cell line (ATCC CRL-1772) and HEK293T cell lines (Thermo Fisher Scientific) were cultured at 37°C in a 5% CO2 incubator with Dulbecco’s modified Eagle medium, supplemented with high glucose (Gibco, Thermo Fisher), 10% fetal bovine serum, 2 mM glutamine and 100 U penicillin/ 0.1 mg/ml streptomycin. Cell lines were purchased with an authentication report prior to purchase.

C2C12 electroporation experiment was carried out by using SE Cell Line 4D-Nucleofector (Lonza) and using the manufacturer’s instructions for 100 ul single Nucleocuvette on the 4D-Nucleofector (Lonza). Plasmid DNA molarity ratio was 0.29 gRNA-297-Lama2: 0.7 Cas9 or GFP.

Lipofectamine 3000 reagent was used to perform the HEK293T transfections following the manufacturer instructions. 240.000 cells were seeded the day before transfection day on a p12 well plate. Plasmid DNA molarity ratio was 0.2 gRNA-TRAC: 0.08 Cas9.

Genomic DNA was extracted using DNeasy Blood and tissue kit (Qiagen) 48 hours post-transfection.

### Library prep and Illumina sequencing for targeted editing analysis

We implemented Illumina sequencing to capture the modified site that was used as our spike-in control with high sensitivity. Plasmids were digested with NotI-HF (NEB) and NheI-HF (NEB) at 37°C for 1h and inactivated at 80°C for 20min; the >750 bps was gel purified using the QIAquick Gel Extraction Kit (Qiagen). A qPCR was performed comparing the CTs of 3727 molecules of edited gDNA and the CTs of the same amount of edited DNA supplemented with different amounts of modified plasmids. Two nested PCRs were performed to the mix that had one lower CT number; using KAPA HiFI DNA Polymerase following manufacturer protocol. We did three different cycles on PCR1 (x25, x30 & x35) and two different concentrations in parallel. PCR1 was done with UMIs-Lama2–271-400_fw and UMIs-Lama2–271-400_rv in a 25 ul final volume, and PCR2 with NEB Index primers and NEB Universal Illumina primers. PCR products were purified with QIAquick PCR Purification Kit (Qiagen), mixed in equimolar ratio, and sequenced with Illumina Miseq Nano kit v2.

### Library prep and Illumina sequencing with Unique Molecular Identifiers (UMIs)

Two nested PCR were performed targeting both the 271-Lama2 and TRAC target sites. The Custom PCR UMI (with SQK-LSK109), version CPU_9107_v109_revA_09Oct2020 (Nanopore Protocol) was followed from UMI tagging step to the late PCR and clean-up step. Unique Molecular Identifiers are added through a PCR of 2 cycles, called UMI tagging, to ensure that each identifier comes just from one molecule. Barcodes to demultiplex by sample are added later, after the UMI tagging, in the early and late PCR. Primers for the UMI tagging were designed using UMI tagging and gene specific primers with amplicon length of around 400 bps. Primers for the early and late PCR were the Illumina Universal primer and Illumina barcoded primer 2 and 4, for 271-Lama2 and TRAC respectively (S7 Table). The purified amplicons were then mixed in equimolar ratio and sequenced with Illumina Miseq Nano kit v2.

## Supporting information

S1 Table Overview of the presence of key features for precision and accessibility within the compared genome editing analysis tools. (ODS)

S2 Table Samples compared in depth in indels benchmarking. (ODS)

S3 Table Samples compared in depth in HDR benchmarking. (JPG)

S4 Table Sample SRR10286522 human validated for HDR benchmarking. (JPG)

S5 Table Sample SRR10285861 human validated for PE benchmarking. (JPG)

S6 Table Samples compared to evaluate distance metric for SimGE evaluation. (JPG)

S7 Table Primers used in this study. (JPG)

S1 Fig Indels distribution. Distribution of insertions (on the left) and deletions (on the right) sizes (on the top) and positions (on the bottom) using the data from the Leenay RT et al. A and Van Overbeek M et al. B studies. In red is the cut-site position. The dataset for the training split 1 is used for both sections of the figure. For the B, the HEK293 was selected from the other two cell lines as all three showed similar patterns.
(PNG)

S2 Fig Heatmap to visualize hierarchical clustering of real samples and their replicates. Data values are transformed to the color scale depicted on the right of the figures. Dark blue equals 0 distance and identical samples, while red is for the greatest distance value. On the top of the figure, we have the heatmaps using Kullback-Leibler distance, while on the bottom we can see the ones using Jensen-Shannon. The plots on the right show the distances between the position of the variant and the cut site, while the plots on the left are for the size of the variants. For these plots, we have selected 5 pairs of real samples from the T cells data set, which have within them the same experimental conditions, same gRNA, and same target. The details for the samples used for these plots are shown in S4 Table.
(PNG)

S3 Fig CRISPR-A diagram. Schema of CRISPR-A pipeline workflow from input to output showing the different alternatives when running the tool and optional processes (in purple). Reads can be simulated, instead of uploaded. After NGS raw data quality control, sequences can be clusterized by UMIs. Reference sequences can be directly uploaded or discovered through the alignment of sequencing reads against a reference genome. Once a reference sequence has the same orientation as gRNA, reads are aligned against it using a mapper with parameters optimized to find indels. Finally, variant calling with error correction is performed and results are shown in multiple plots and tables. B) Parameters fitted in gene editing simulator (SimGE). We have calculated the probability matrix for each kind of mutation in the function of the different parameters (nucleotide composition, distance to cut, and modification size). We have also used information related to micro-homology patterns and epigenetic data to get the ratio between NHEJ and MMEJ events. C) Simulation engine. Cell line information and sequence context are conditionants of the percentage of different events. SimGE works with different layers: First, the proportion of edited and wild type sequences is determined. Second, the proportion of each kind of modification (substitutions, deletions, and insertions) is determined. Third, the proportion of subtypes of modifications is sampled from density probabilities distribution functions fitted with the training data set.
(PNG)

S4 Fig Total count of sequences that have not aligned or that have alignment giving rise to incorrect characterizations by different alignment methods. (PNG)

S5 Fig Percentage reported by CRISPR-A against the percentage of edition reported by other tools. CRISPR-GA is also compared after merging R1 and R2 reads, and CRISPResso2 is also shown when the quantification window is used as default. (PNG)

S6 Fig IGV graphical representation of the alignment of reads aligning against the amplicon reference from the reference genome or with the same amplicon reference including the inversion observed in the previous alignment (natural variant enhancing confusion). (PNG)

S7 Fig IGV graphical representation of the alignment of reads aligning against a sequence shorter than the amplicon reference and against the whole amplicon reference. (PNG)

S8 Fig IGV graphical representation of the alignment of reads aligning against the amplicon reference. A) A polyadenine of 14 nucleotides is poorly sequenced adding a pick of indels far from the cut site. B) Results after ignoring error prone regions.
(JPG)

S9 Fig Percentage of each spike-in among the total count of reads characterized as spik-in molecules using different amounts of initial sike-in molecules and with a different number of PCR cycles. (PNG)

S10 Fig Exploration of identity and UBS of clusters. A) Percentage of insertion and deletions equal or different from the one characterized in the consensus sequence of the cluster by UMI bin size (UBS) and target and clustering identity. B) Noise reduction by cluster filtering (identity: 0.95; UBS>5 and UBS<130) in TRAC locus. The red dashed line corresponds to the cut site position.
(PNG)

S11 Fig Differences between mock and treated feature distributions. A) Distributions of the mean distance to the cut site from mock files (gray) versus treated files (colored). B) Distributions of median indel length in bp’s from mock files (gray) versus treated files (colored). C) Distributions of average Insertion/deletion frequency from mock files (gray) and treated files (colored). All the replicates per cell line were assessed for normality. The differences between the distributions evaluated are between the population of noise events (mock files) and a mixed population of noise+real edits, in which the last one is the most represented, thus being able to appreciate the differences between them.
(PNG)

S12 Fig Distance from editing percentage reporter by different gene editing analysis tools and the edition percentage established in the human validation process. Samples are sorted from left to right by average distance, being CRISPECTOR the tool with minimal mean distance and CRISPRpic the tool with higher mean distance to the human determined percentage of edition.
(PNG)

S13 Fig Representativity, efficiency, and sensitivity assessment of the aligned depth normalized, stratified, mock vs treatment comparison model. A) Representativity of mock events within their respective treated files. B) Representativity of indel events within their respective mock files in percentage (%). C) Correction efficiency without normalizing per aligned depth. D) Correction efficiency normalizing per aligned depth. The representativity of all events was measured, stratifying by position size and indel type.
(PNG)

S14 Fig Correction deep exploration. The plots correspond to an old version of CRISPR-A, produced to have a better taste of the differences discussed when comparing our tool with CRISPECTOR. In SRR3698365 the WARNING message about low aligned depth is shown at the top.
(PNG)

S15 Fig Correlation between different features and representativity. A) Correlation between the number of aligned reads of treated files and representativity. B) Correlation between the number of events of treated files and representativity. C) Correlation between the number of aligned reads of mock files and representativity. D) Correlation between the number of events of mock files and representativity.
(PNG)

S16 Fig PCAs of alignment parameters Monte Carlo and accuracy of their combinations, from PCA1 to PCA4. (JPG)

S1 Video CRISPR-A web overview. (MOV)
