# Supplementary figures and images for: CRISPR-Analytics (CRISPR-A): A platform for precise analytics and simulations for gene editing
Source: PLoS Comput Biol. 2023 May 30;19(5):e1011137. doi: 10.1371/journal.pcbi.1011137 (PMC10256225; doi:10.1371/journal.pcbi.1011137)

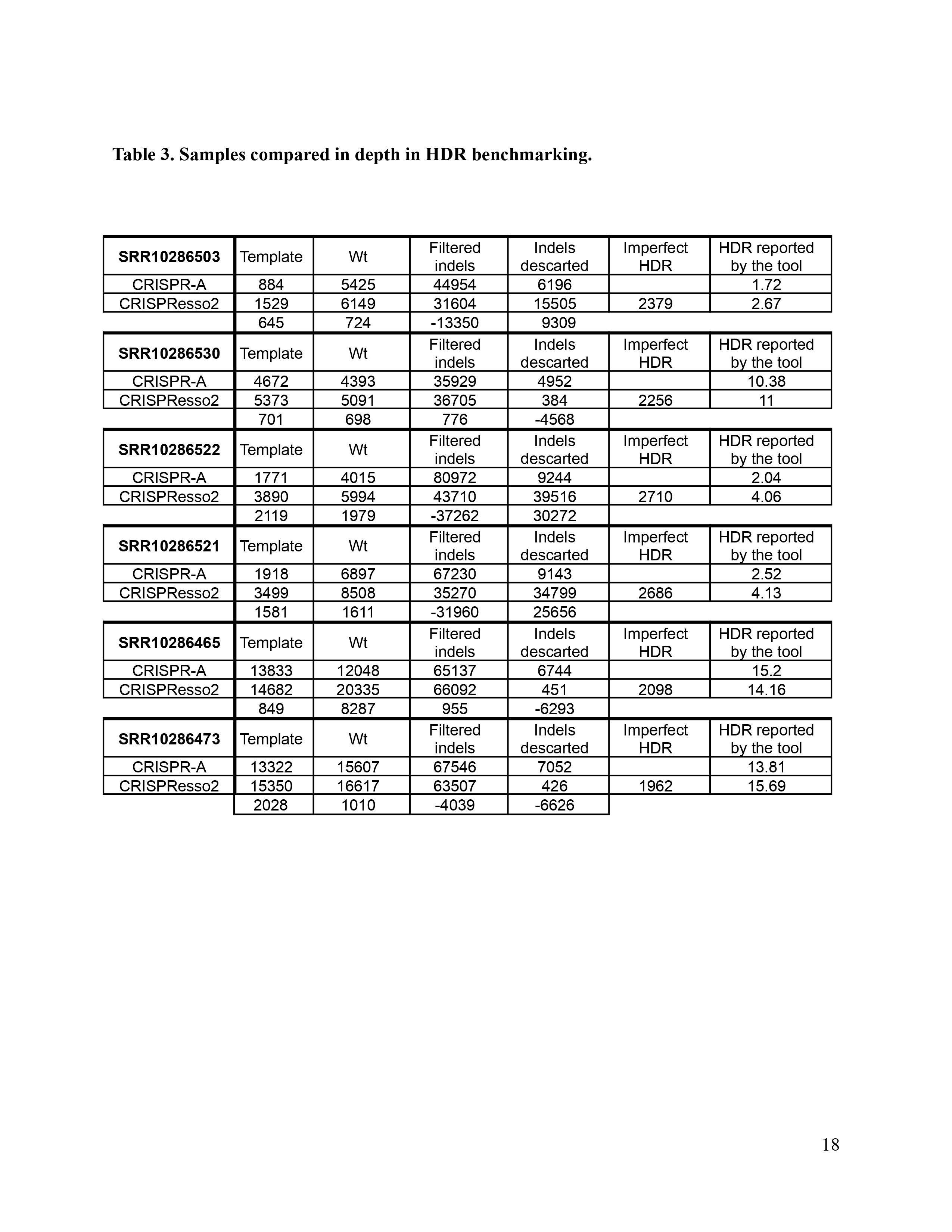

Supplement: S3 Table — (JPG) [file pcbi.1011137.s003.jpg]

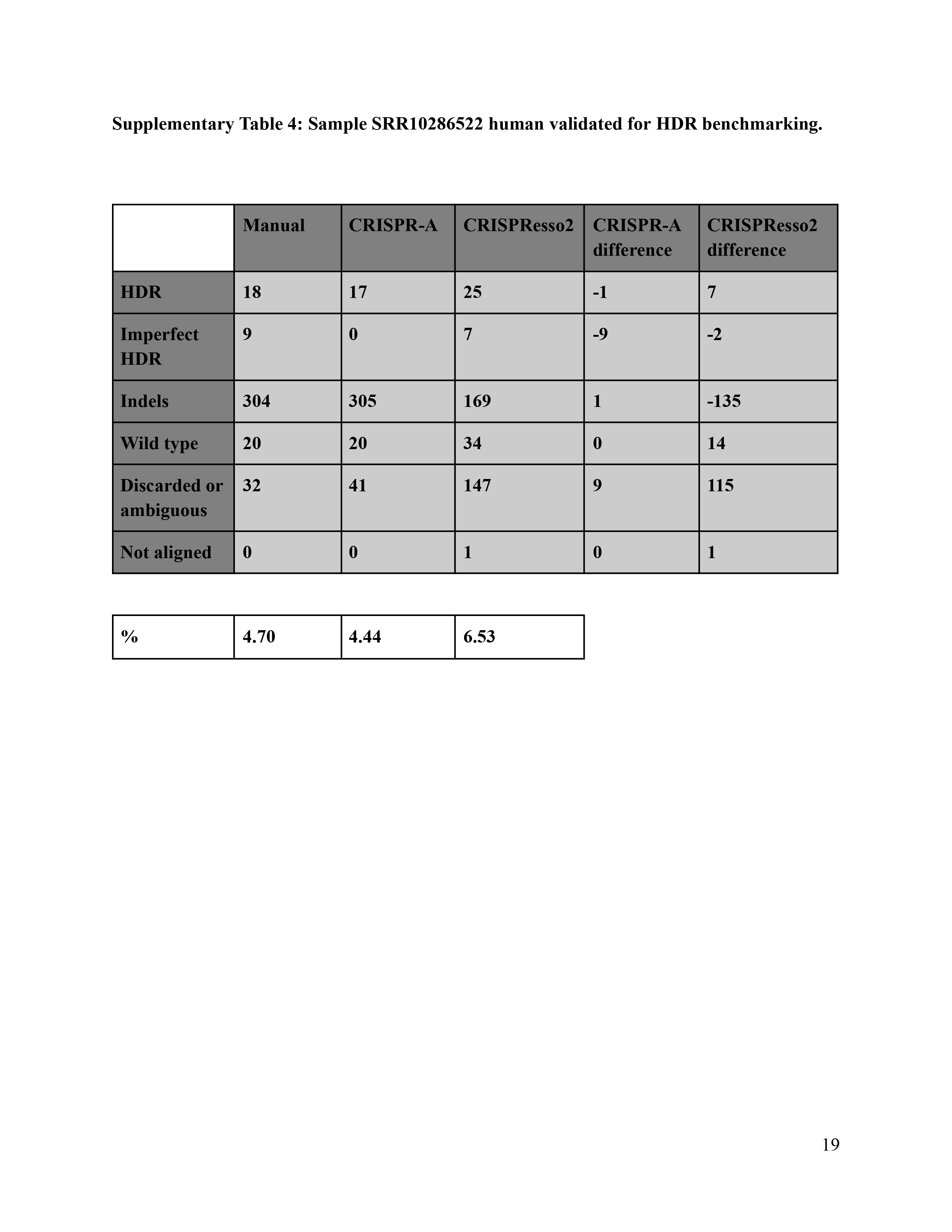

Supplement: S4 Table — (JPG) [file pcbi.1011137.s004.jpg]

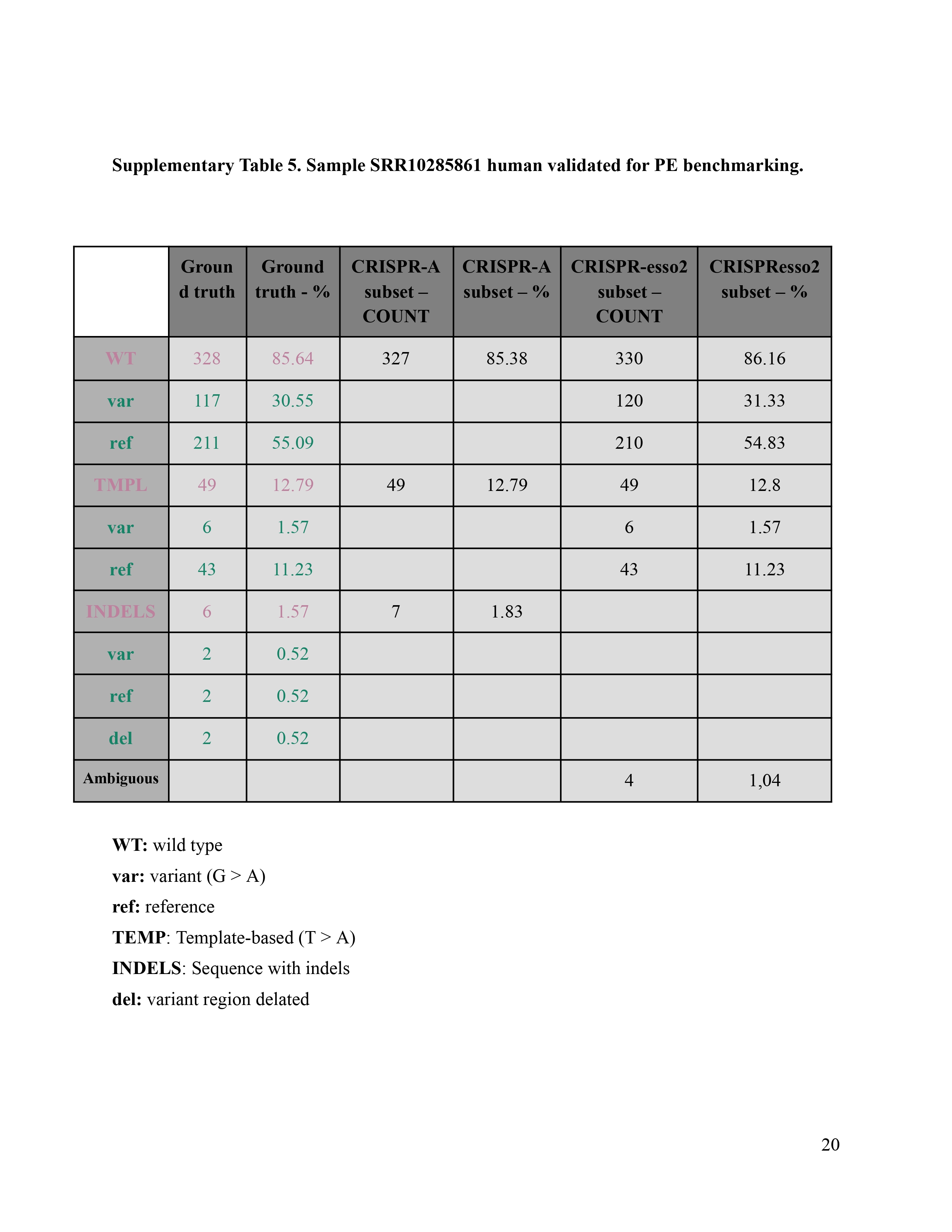

Supplement: S5 Table — (JPG) [file pcbi.1011137.s005.jpg]

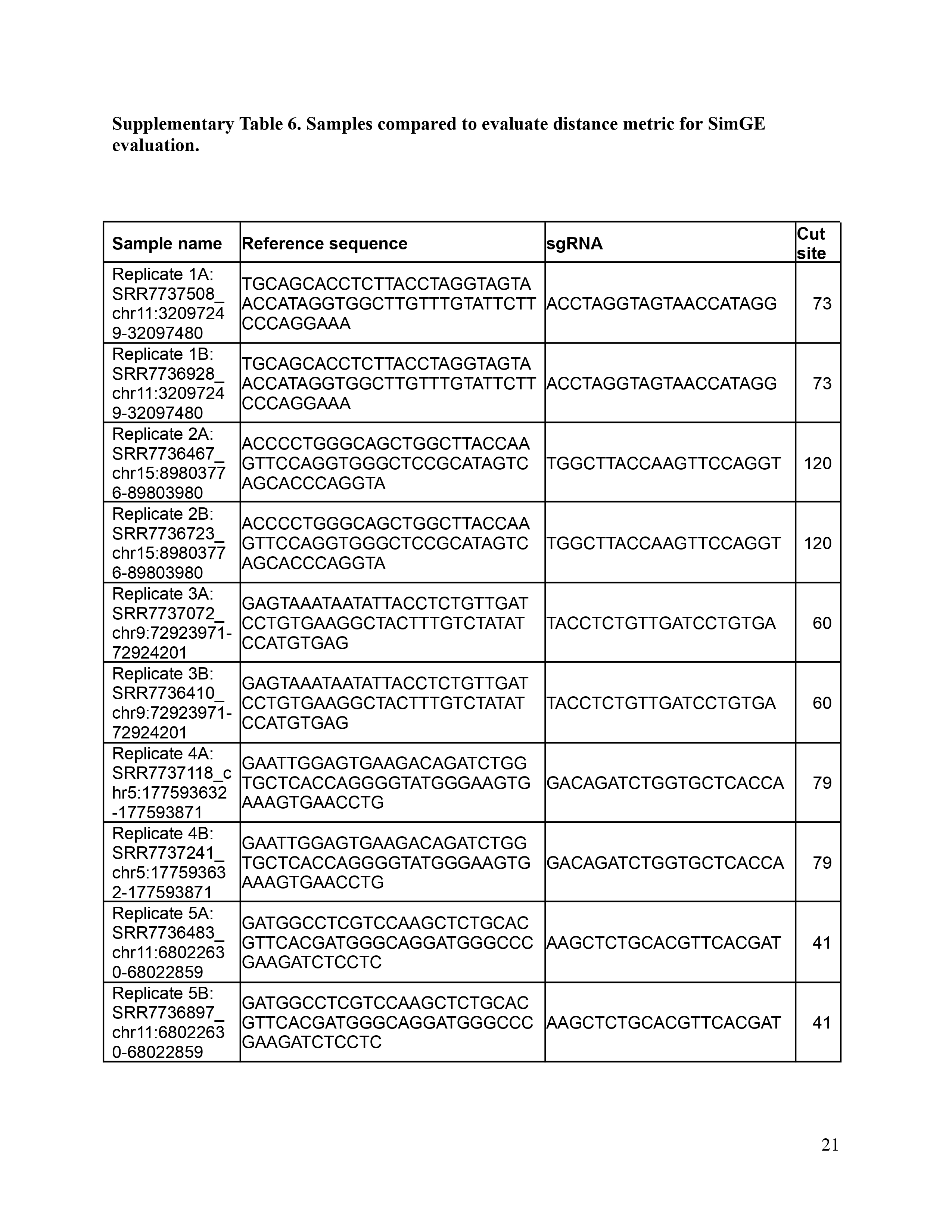

Supplement: S6 Table — (JPG) [file pcbi.1011137.s006.jpg]

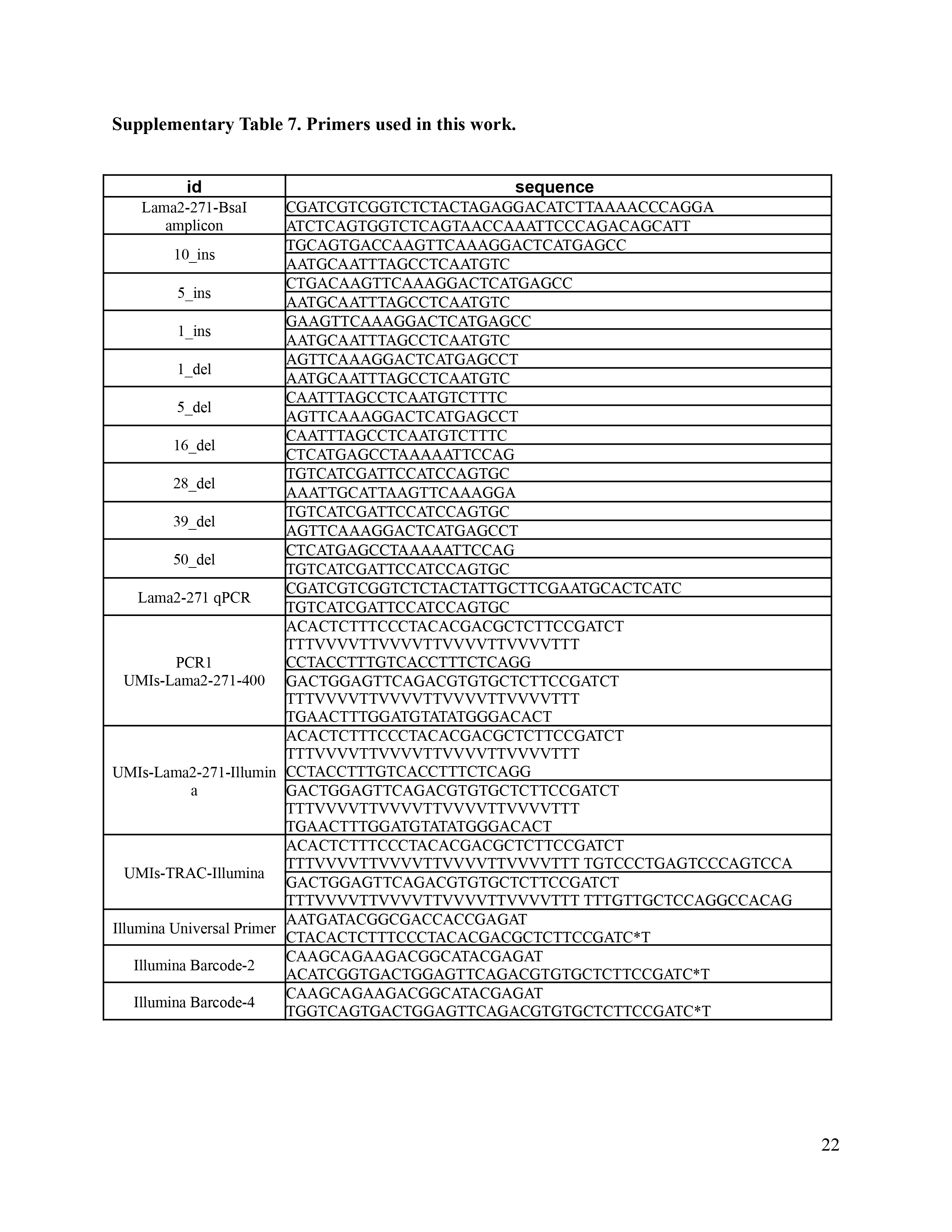

Supplement: S7 Table — (JPG) [file pcbi.1011137.s007.jpg]

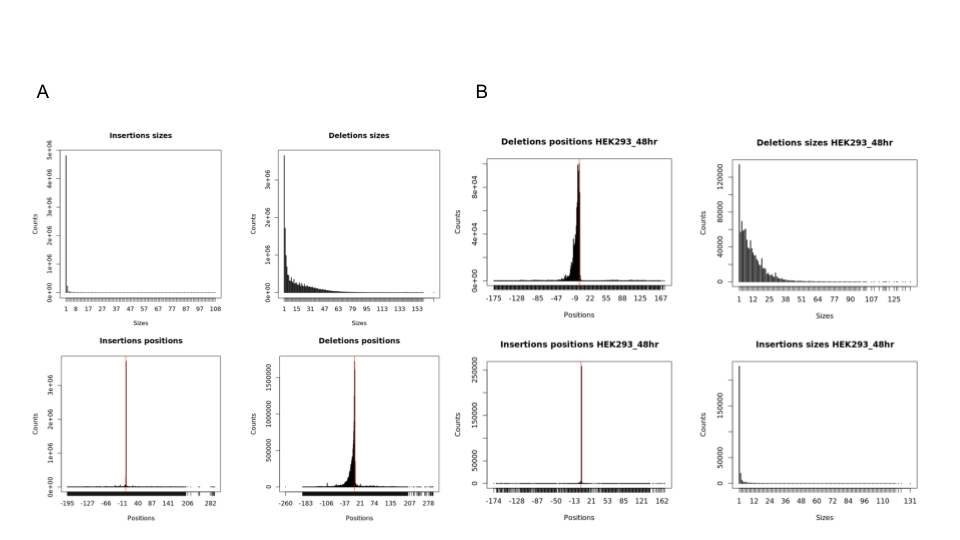

Supplement: S1 Fig — Distribution of insertions (on the left) and deletions (on the right) sizes (on the top) and positions (on the bottom) using the data from the Leenay RT et al. A and Van Overbeek M et al. B studies. In red is the cut-site position. The dataset for the training split 1 is used for both sections of the figure. For the B, the HEK293 was selected from the other two cell lines as all three showed similar patterns. (PNG) [file pcbi.1011137.s008.png]

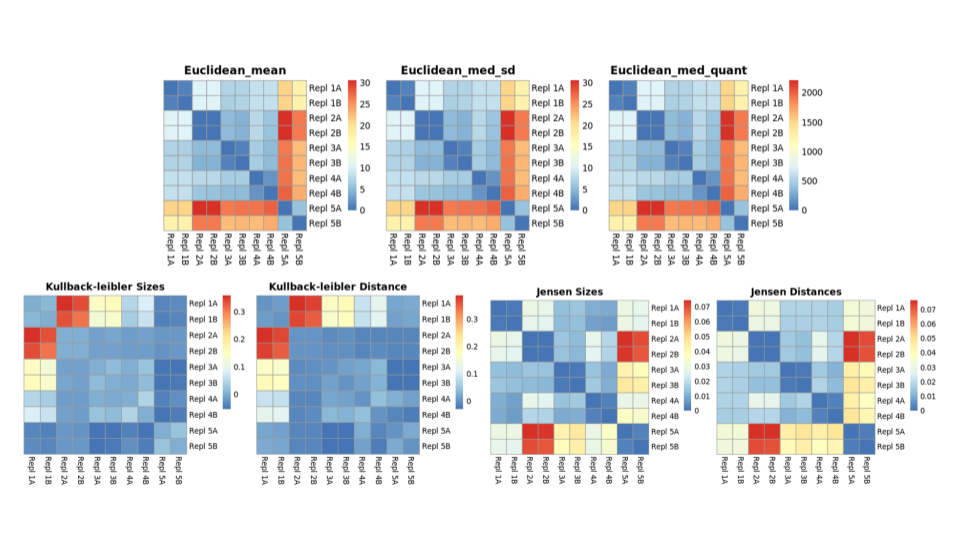

Supplement: S2 Fig — Data values are transformed to the color scale depicted on the right of the figures. Dark blue equals 0 distance and identical samples, while red is for the greatest distance value. On the top of the figure, we have the heatmaps using Kullback-Leibler distance, while on the bottom we can see the ones using Jensen-Shannon. The plots on the right show the distances between the position of the variant and the cut site, while the plots on the left are for the size of the variants. For these plots, we have selected 5 pairs of real samples from the T cells data set, which have within them the same experimental conditions, same gRNA, and same target. The details for the samples used for these plots are shown in S4 Table. (PNG) [file pcbi.1011137.s009.png]

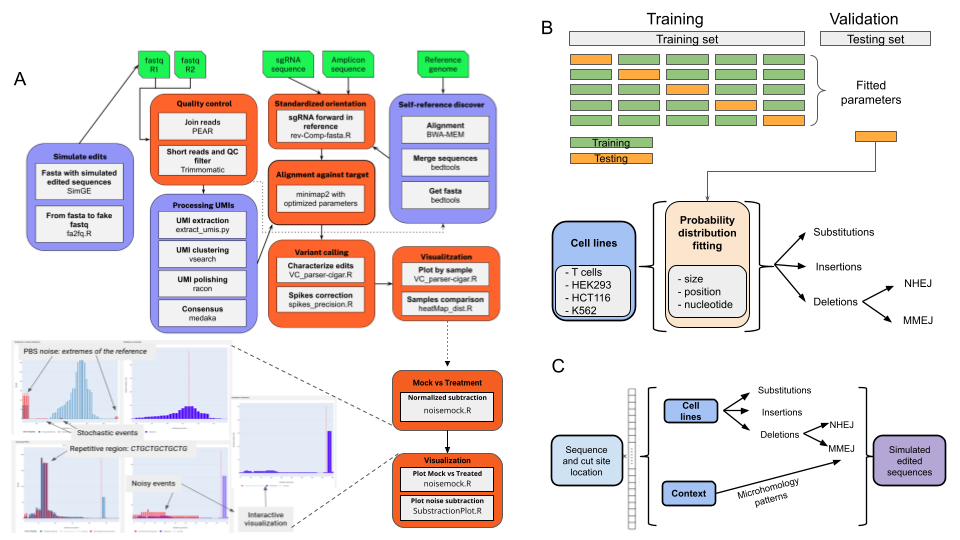

Supplement: S3 Fig — Schema of CRISPR-A pipeline workflow from input to output showing the different alternatives when running the tool and optional processes (in purple). Reads can be simulated, instead of uploaded. After NGS raw data quality control, sequences can be clusterized by UMIs. Reference sequences can be directly uploaded or discovered through the alignment of sequencing reads against a reference genome. Once a reference sequence has the same orientation as gRNA, reads are aligned against it using a mapper with parameters optimized to find indels. Finally, variant calling with error correction is performed and results are shown in multiple plots and tables. B) Parameters fitted in gene editing simulator (SimGE). We have calculated the probability matrix for each kind of mutation in the function of the different parameters (nucleotide composition, distance to cut, and modification size). We have also used information related to micro-homology patterns and epigenetic data to get the ratio between NHEJ and MMEJ events. C) Simulation engine. Cell line information and sequence context are conditionants of the percentage of different events. SimGE works with different layers: First, the proportion of edited and wild type sequences is determined. Second, the proportion of each kind of modification (substitutions, deletions, and insertions) is determined. Third, the proportion of subtypes of modifications is sampled from density probabilities distribution functions fitted with the training data set. (PNG) [file pcbi.1011137.s010.png]

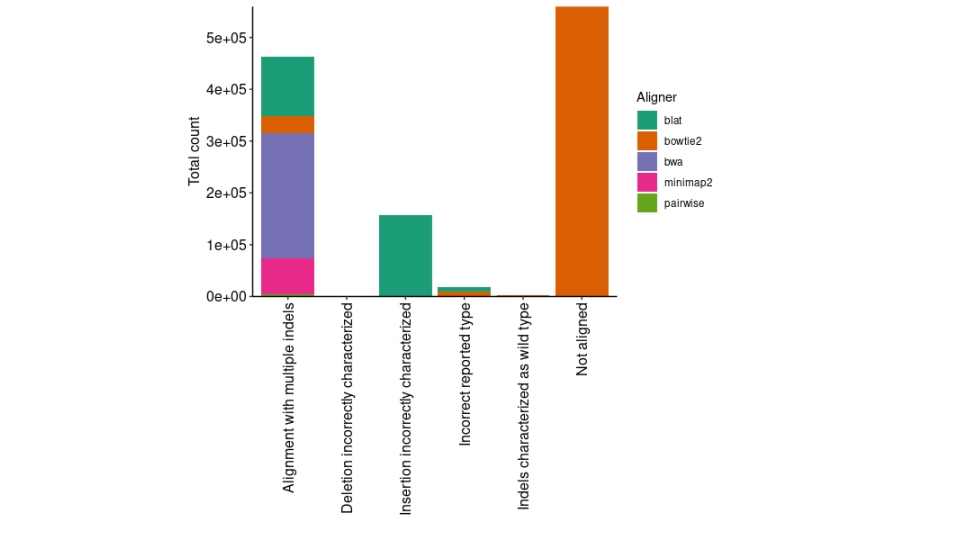

Supplement: S4 Fig — (PNG) [file pcbi.1011137.s011.png]

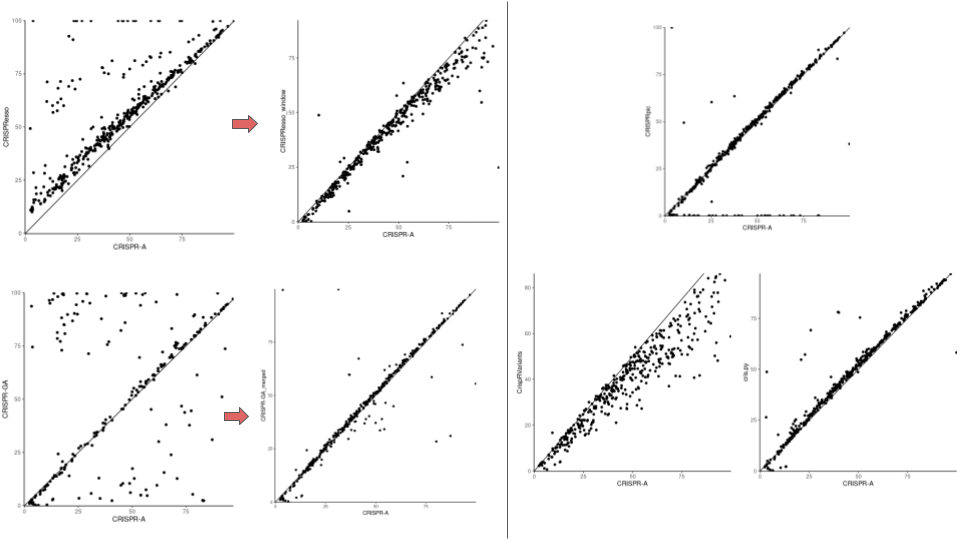

Supplement: S5 Fig — (PNG) [file pcbi.1011137.s012.png]

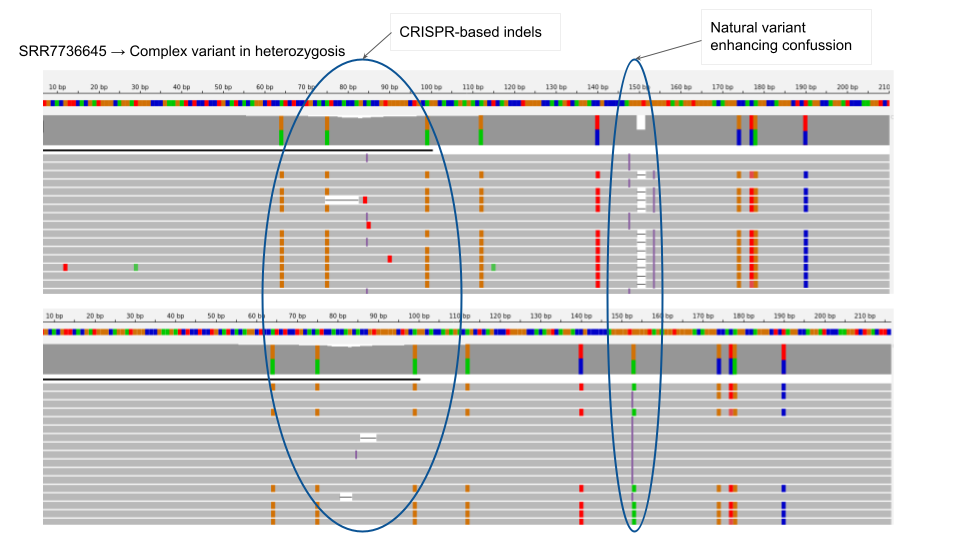

Supplement: S6 Fig — (PNG) [file pcbi.1011137.s013.png]

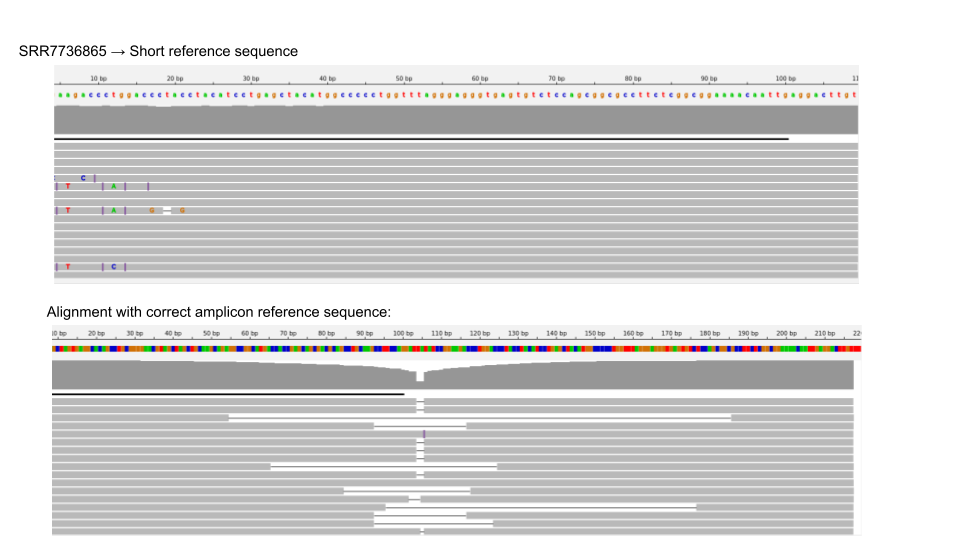

Supplement: S7 Fig — (PNG) [file pcbi.1011137.s014.png]

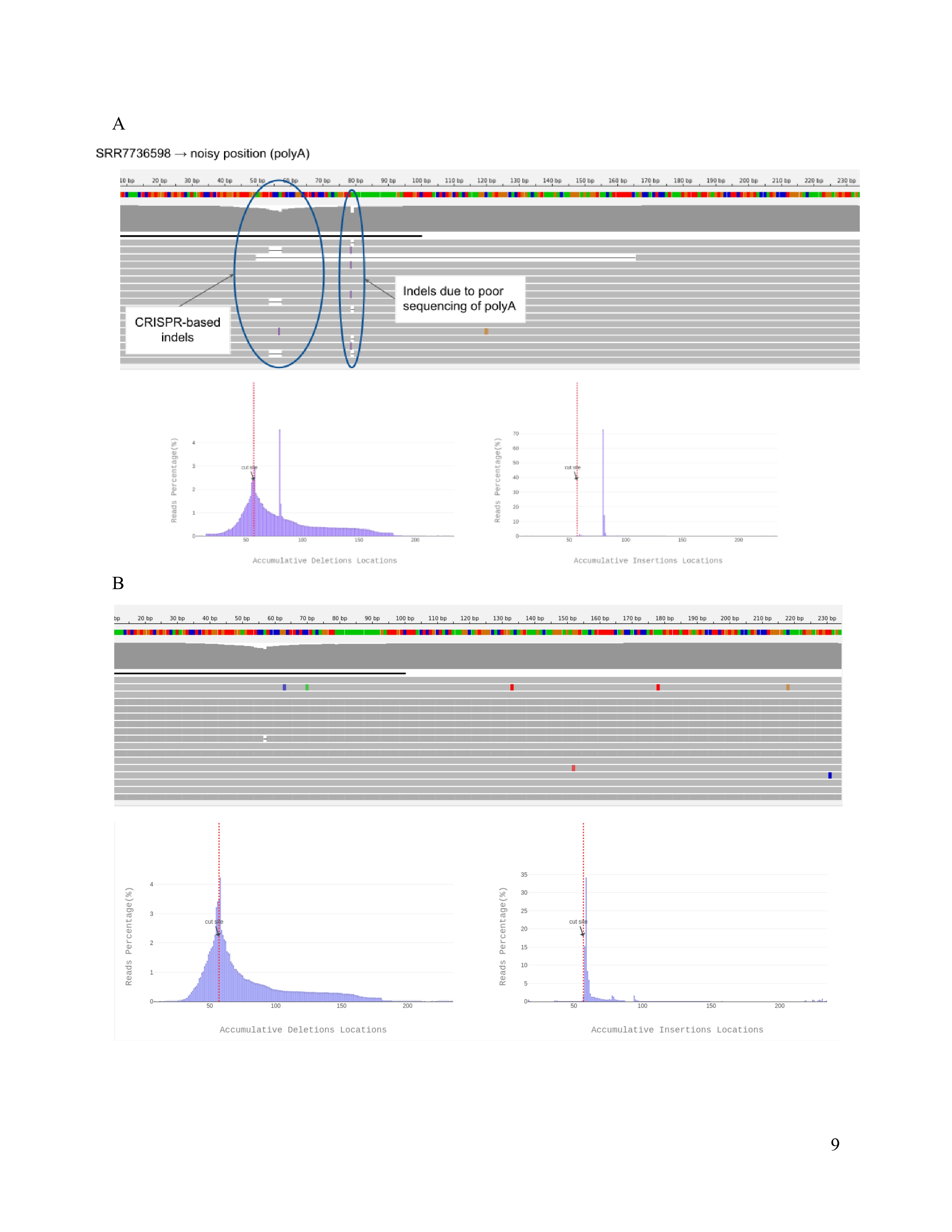

Supplement: S8 Fig — A) A polyadenine of 14 nucleotides is poorly sequenced adding a pick of indels far from the cut site. B) Results after ignoring error prone regions. (JPG) [file pcbi.1011137.s015.jpg]

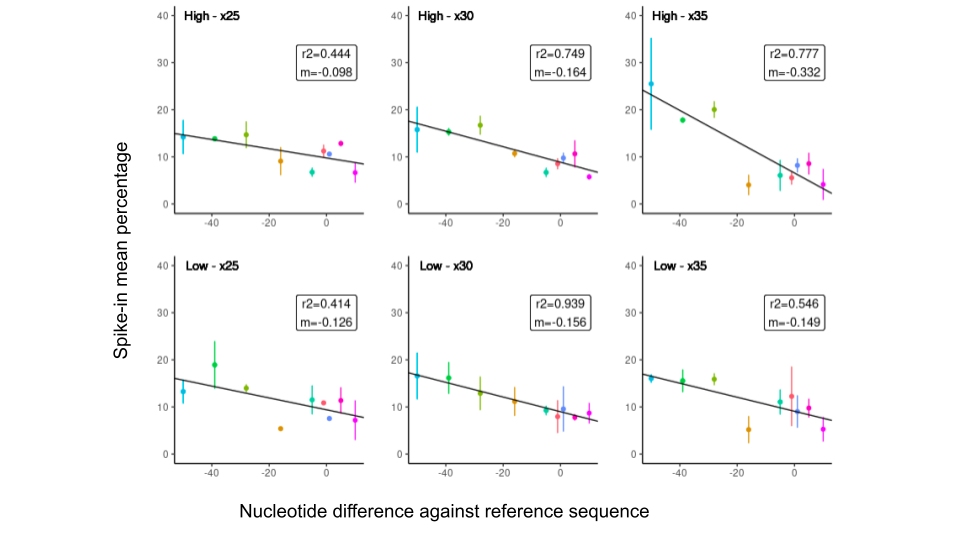

Supplement: S9 Fig — (PNG) [file pcbi.1011137.s016.png]

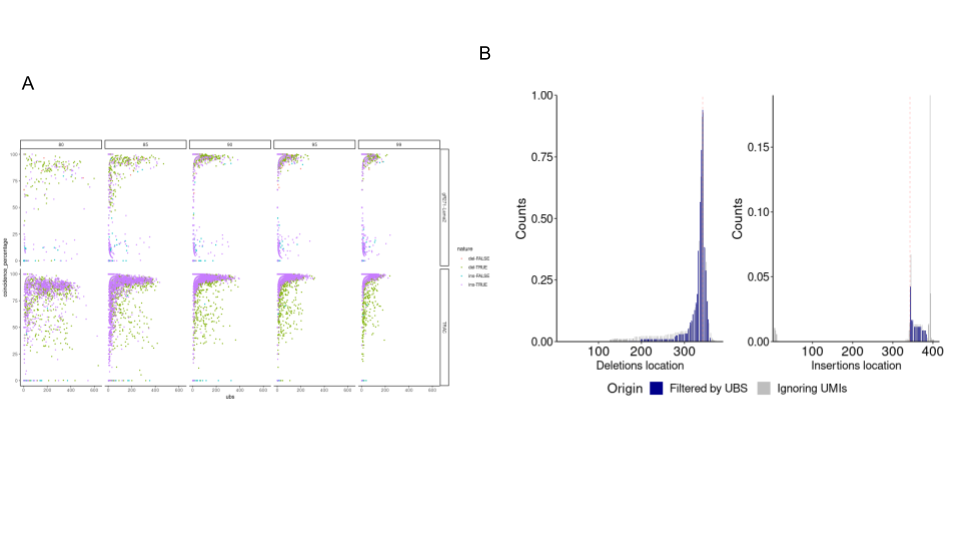

Supplement: S10 Fig — A) Percentage of insertion and deletions equal or different from the one characterized in the consensus sequence of the cluster by UMI bin size (UBS) and target and clustering identity. B) Noise reduction by cluster filtering (identity: 0.95; UBS>5 and UBS<130) in TRAC locus. The red dashed line corresponds to the cut site position. (PNG) [file pcbi.1011137.s017.png]

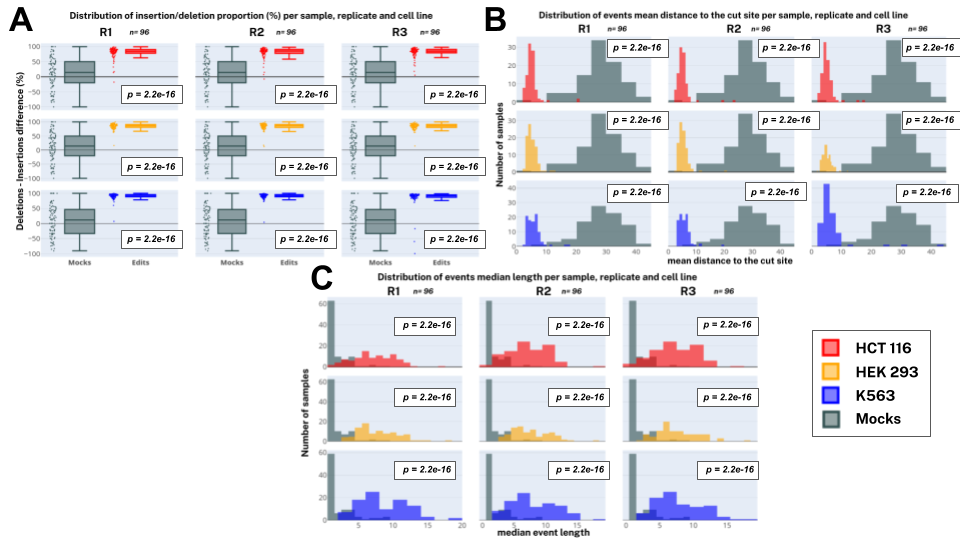

Supplement: S11 Fig — A) Distributions of the mean distance to the cut site from mock files (gray) versus treated files (colored). B) Distributions of median indel length in bp’s from mock files (gray) versus treated files (colored). C) Distributions of average Insertion/deletion frequency from mock files (gray) and treated files (colored). All the replicates per cell line were assessed for normality. The differences between the distributions evaluated are between the population of noise events (mock files) and a mixed population of noise+real edits, in which the last one is the most represented, thus being able to appreciate the differences between them. (PNG) [file pcbi.1011137.s018.png]

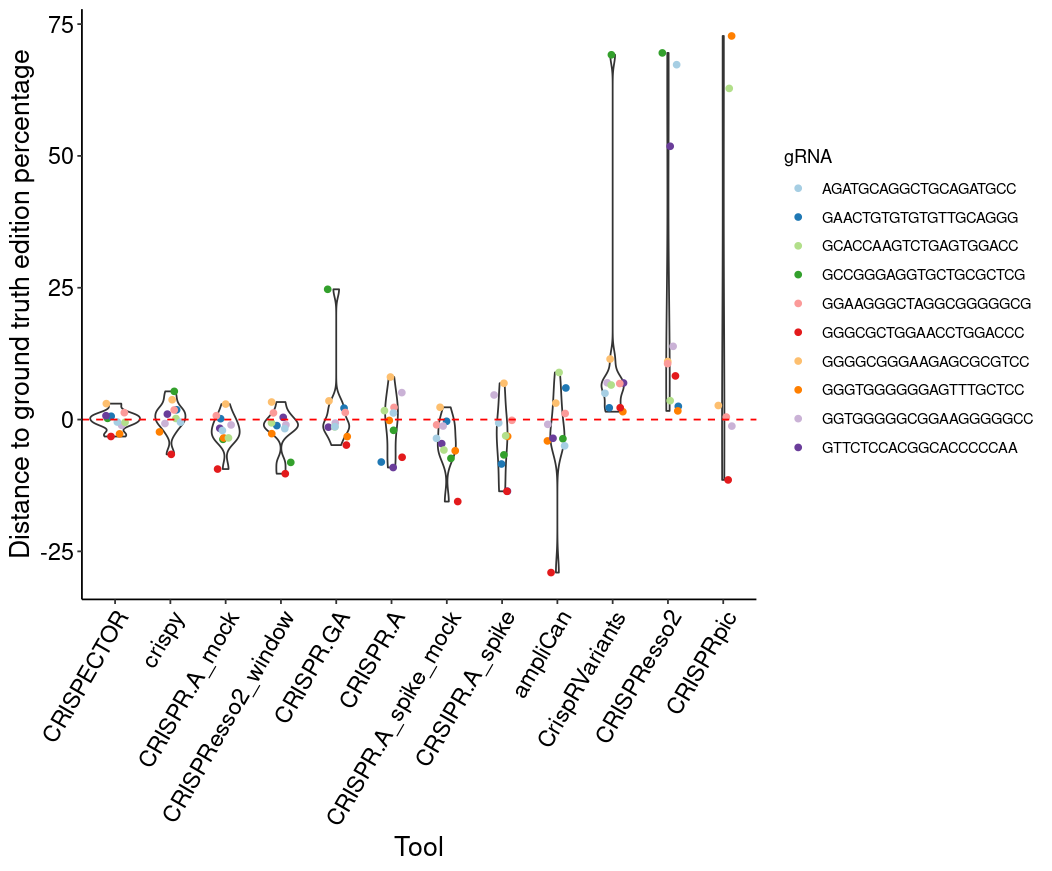

Supplement: S12 Fig — Samples are sorted from left to right by average distance, being CRISPECTOR the tool with minimal mean distance and CRISPRpic the tool with higher mean distance to the human determined percentage of edition. (PNG) [file pcbi.1011137.s019.png]

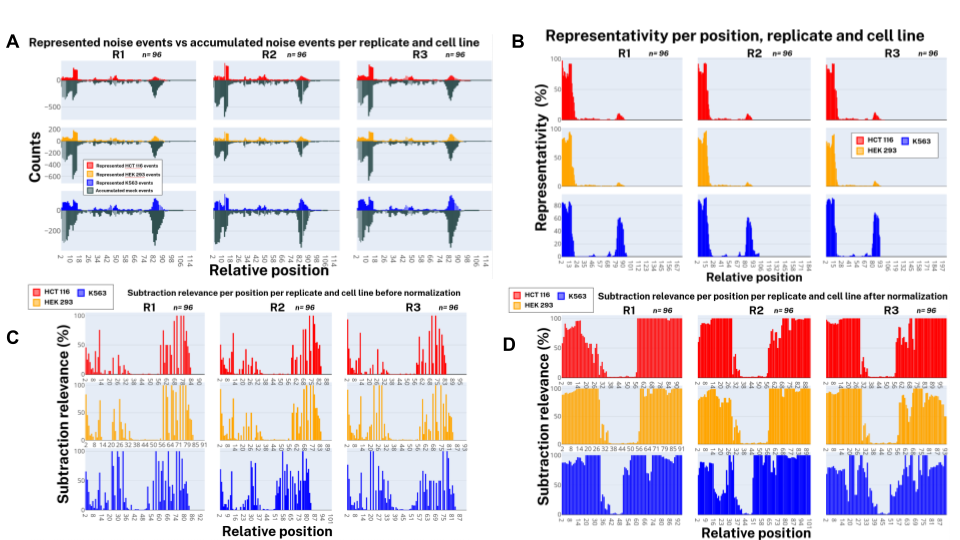

Supplement: S13 Fig — A) Representativity of mock events within their respective treated files. B) Representativity of indel events within their respective mock files in percentage (%). C) Correction efficiency without normalizing per aligned depth. D) Correction efficiency normalizing per aligned depth. The representativity of all events was measured, stratifying by position size and indel type. (PNG) [file pcbi.1011137.s020.png]

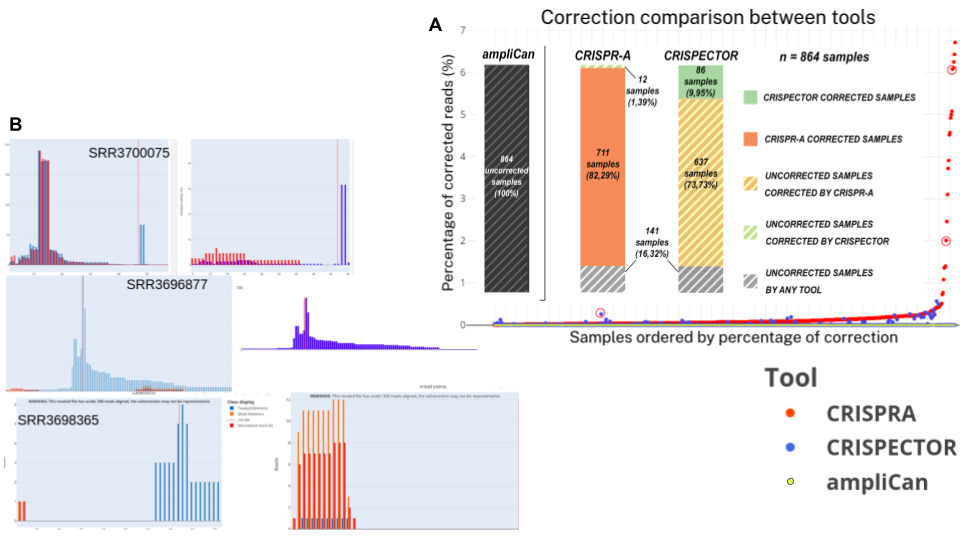

Supplement: S14 Fig — The plots correspond to an old version of CRISPR-A, produced to have a better taste of the differences discussed when comparing our tool with CRISPECTOR. In SRR3698365 the WARNING message about low aligned depth is shown at the top. (PNG) [file pcbi.1011137.s021.png]

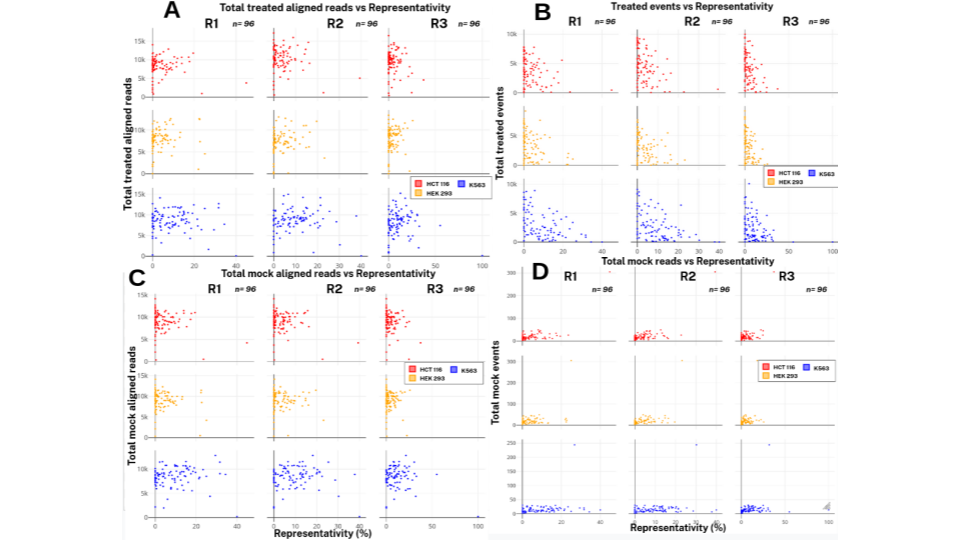

Supplement: S15 Fig — A) Correlation between the number of aligned reads of treated files and representativity. B) Correlation between the number of events of treated files and representativity. C) Correlation between the number of aligned reads of mock files and representativity. D) Correlation between the number of events of mock files and representativity. (PNG) [file pcbi.1011137.s022.png]

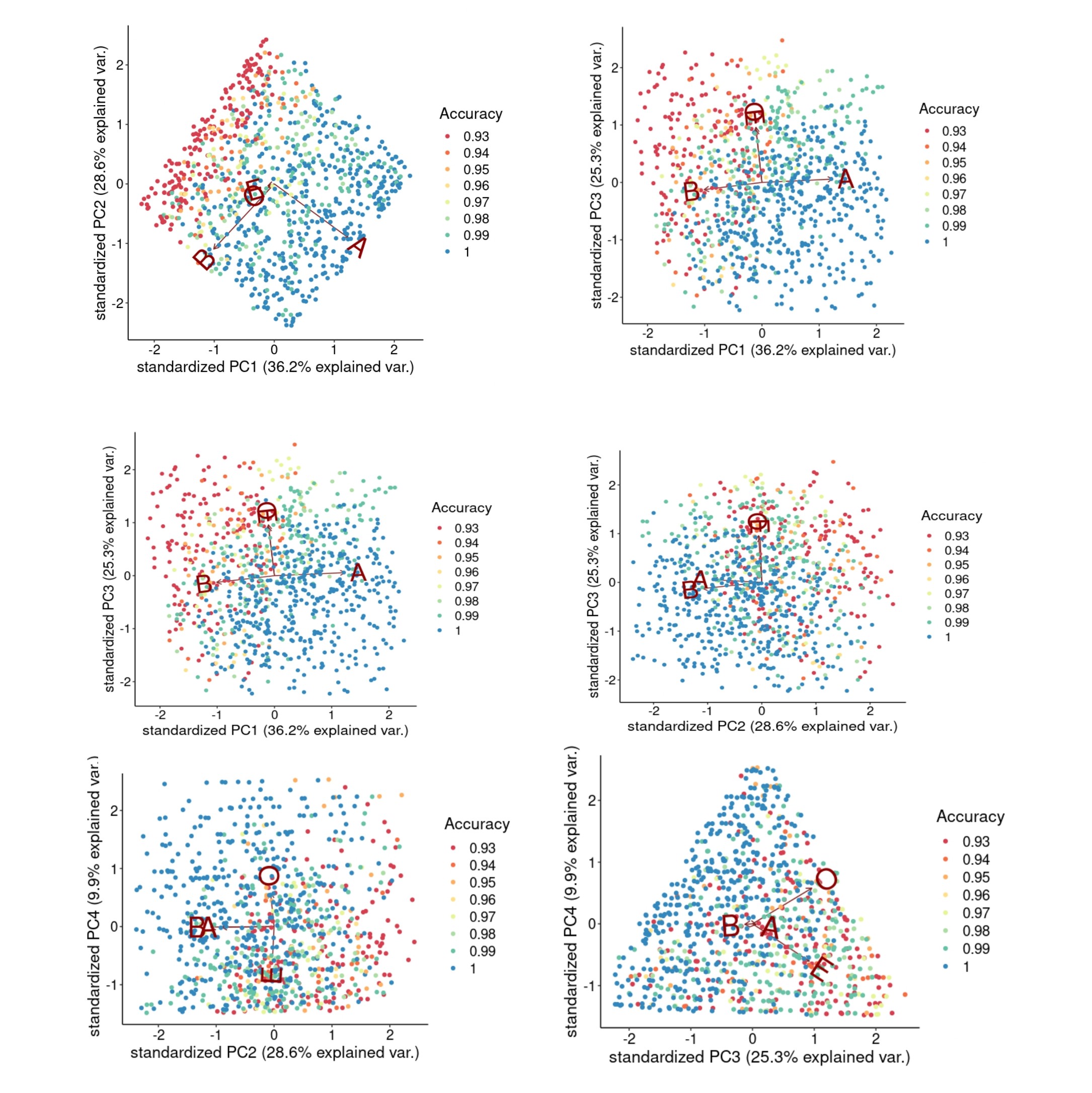

Supplement: S16 Fig — (JPG) [file pcbi.1011137.s023.jpg]
